# Initial COVID-19 severity influenced by SARS-CoV-2-specific T cells imprints T-cell memory and inversely affects reinfection

**DOI:** 10.1038/s41392-024-01867-4

**Published:** 2024-05-29

**Authors:** Gang Yang, Jinpeng Cao, Jian Qin, Xinyue Mei, Shidong Deng, Yingjiao Xia, Jun Zhao, Junxiang Wang, Tao Luan, Daxiang Chen, Peiyu Huang, Cheng Chen, Xi Sun, Qi Luo, Jie Su, Yunhui Zhang, Nanshan Zhong, Zhongfang Wang

**Affiliations:** 1 The Affiliated Hospital of Kunming University of Science and Technology. Department of Respiratory and Critical Care Medicine, The First People’s Hospital of Yunnan province, Kunming, Yunnan China; 2 Guangzhou National Laboratory, Bioland, Guangzhou, Guangdong China; 3 State Key Laboratory of Respiratory Disease & National Clinical Research Center for Respiratory Disease, Guangzhou Institute of Respiratory Health, the First Affiliated Hospital of Guangzhou Medical University, Guangzhou Medical University, Guangzhou, China

**Keywords:** Adaptive immunity, Infectious diseases, Respiratory tract diseases, Infectious diseases

## Abstract

The immunoprotective components control COVID-19 disease severity, as well as long-term adaptive immunity maintenance and subsequent reinfection risk discrepancies across initial COVID-19 severity, remain unclarified. Here, we longitudinally analyzed SARS-CoV-2-specific immune effectors during the acute infection and convalescent phases of 165 patients with COVID-19 categorized by severity. We found that early and robust SARS-CoV-2-specific CD4^+^ and CD8^+^ T cell responses ameliorate disease progression and shortened hospital stay, while delayed and attenuated virus-specific CD8^+^ T cell responses are prominent severe COVID-19 features. Delayed antiviral antibody generation rather than titer level associates with severe outcomes. Conversely, initial COVID-19 severity imprints the long-term maintenance of SARS-CoV-2-specific adaptive immunity, demonstrating that severe convalescents exhibited more sustained virus-specific antibodies and memory T cell responses compared to mild/moderate counterparts. Moreover, initial COVID-19 severity inversely correlates with SARS-CoV-2 reinfection risk. Overall, our study unravels the complicated interaction between temporal characteristics of virus-specific T cell responses and COVID-19 severity to guide future SARS-CoV-2 wave management.

## Introduction

Adaptive immunity is crucial in combating viral infections and facilitating recovery from severe acute respiratory syndrome coronavirus 2 (SARS-CoV-2) infection, thereby influencing the disease severity of Coronavirus disease 2019 (COVID-19).^1,2^ During the acute infection phase, previous studies suggested that the reduced disease severity and effective virus clearance are related to coordinated anti-SARS-CoV-2 adaptive immunity.^3,4^ Delayed neutralizing antibody (Nab) production correlates with COVID-19 mortality, whereas higher antibody titers during later infection in severe and fatal cases do not affect the amelioration of disease severity.^3,5^ In mild infection cases, the frequencies of early nucleocapsid-specific CD4^+^ T cells^6^ and activated spike-specific CD8^+^ T cells^7^ negatively correlate with upper respiratory viral loads. In contrast, patients with severe COVID-19 exhibit broad lymphopenia and extensive functional impairment in CD4^+^ and CD8^+^ T cell subsets.^8^

During the memory immunity phase, Jung et al. has evaluated long-term SARS-CoV-2-specific T-cell memory more than 200 days post symptom onset (dps) by ELISpot and reported similar levels between asymptomatic/mild and severe/critical patients.^9^ While Peng et al. found that early convalescent ( ~ 40 dps) individuals who recovered from severe COVID-19 relative to those with mild infection had significantly higher breadth and robustness of memory T cell responses.^10^ Two studies indicated that the tested timepoints of T cell response will also influence the magnitude and durability of adaptive immune memory. Therefore, a thorough study across acute, recovery and memory phase between different severity will be necessary.^9–12^ Moreover, the lack of a clear association between memory immunity and disease severity further complicates the understanding between COVID-19 severity and SARS-CoV-2 reinfection risk. Current studies primarily suggest the significant decline in the Nab titers over time may be linked to the risk of SARS-CoV-2 reinfection.^13–15^ However, in real-world observation, even when the Nab titers dropped to similar levels, individuals may still exhibit different reinfection risk, suggesting the presence of other memory immune effectors beyond Nab that can influence SARS-CoV-2 reinfection. Therefore, elucidating the interaction between the initial COVID-19 severity and long-term adaptive immune responses, as well as the subsequent risk of reinfection, is imperative for effective management of future infection wave.

In the present study, we established a longitudinal cohort with varying COVID-19 severities during the Omicron BA.5 wave in China, and collected serial and paired samples throughout the acute infection and recovery (4- and 7-months post-recovery) phases. The viral loads in the upper respiratory tract were quantified, and the acute and memory SARS-CoV-2-specific CD4^+^ and CD8^+^ T cell responses together with the antiviral antibody, were assessed at multiple time-points. The real-world reinfection risks and immune recall responses were investigated. Based on this unique cohort with long observation periods and reinfections, we aimed to systematically dissect the relationship between acute immune effectors and COVID-19 trajectories. Subsequently, we sought to inversely determine the influence of initial severity on the development and persistence of immune memory and subsequent reinfection risk.

## Result

### Patient demographics, clinical characteristics, and viral kinetics

**Fig. 1:**
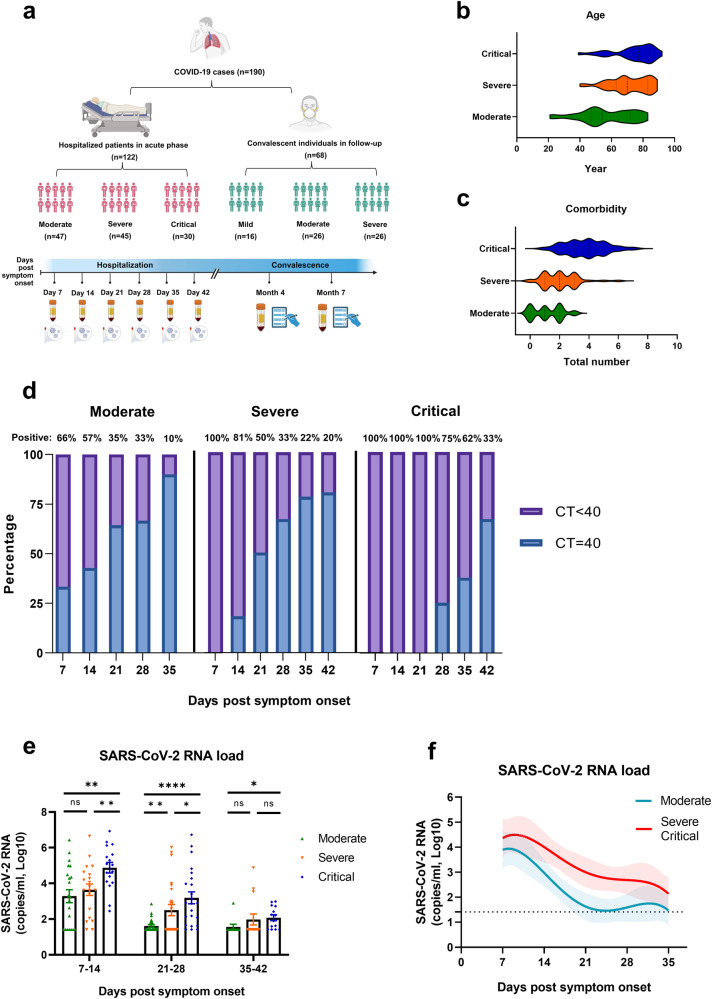
Longitudinal COVID-19 cohort and dynamics of viral load. **a** Schematic of study participants split by severity categories, and longitudinal sample collection including every 7 days during acute COVID-19 symptom onset and at 4- and 7-months post-recovery. **b**, **c** Distribution of age and number of comorbidities between moderate (*n* = 47), severe (*n* = 45) and critical (*n* = 30) groups. **d** Proportion of patients with positive (purple) and negative (blue) nucleic acid tests, divided by a CT value threshold of 40, among the three groups (moderate, 7 dps *n* = 7, 14 dps *n* = 14, 21 dps *n* = 14, 28 dps *n* = 13, 35 dps *n* = 10; severe, 7 dps *n* = 9, 14 dps *n* = 11, 21 dps *n* = 12, 28 dps *n* = 12, 35 dps *n* = 9, 42 dps *n* = 5 ; critical, 7 dps *n* = 6, 14 dps *n* = 11, 21 dps *n* = 10, 28 dps *n* = 12, 35 dps *n* = 8, 42 dps *n* = 6). **e** Comparison of SARS-CoV-2 viral loads in the moderate, severe, and critical groups in the early (7–14 dps), middle (21–28 dps), and late (35–42 dps) stages of acute infection performed by t-test. **f** The kinetics of SARS-CoV-2 RNA clearance among patient groups using a polynomial nonlinear regression model with 95% confidence intervals visually represented by the shaded colors. The dashed line represents the threshold for the virus load at 0. Gender, comorbidity, clinical outcomes between groups were assessed using chi-square test. Each dot represents one donor. *P < 0.05, **P < 0.01, ***P < 0.001

To establish the longitudinal cohort, we recruited 122 hospitalized patients with PCR-confirmed acute SARS-CoV-2 infection (47 moderate, 45 severe, and 30 critical) during the Omicron BA.5 wave in China (Fig. 1a), and 68 recovered individuals (16 mild, 26 moderate, and 26 severe) who participated in the follow-up at 4 months and 7 months during convalescence with an overlap of 25 individuals with the acute hospitalized patients. Analysis of the electronic medical data of the hospitalized COVID-19 patients revealed that disease severity worsened as the age and male proportion increased (*P* < 0.0001) (Fig. 1b), resulting in a significant increase in the duration of hospital stay (Supplementary Table 1), consistent with previous reports.^16,17^ The total number of comorbidities (Fig. 1c) and the prevalence of underlying cardiovascular conditions increased significantly in the critical group (*P* < 0.0001) (Supplementary Table 1), which may further exacerbate COVID-19 severity. Additionally, up to 53% of critically ill patients had co-existing bacterial or fungal infections during the hospitalization period, with a prevalence of 26% in severe patients versus only 8% in moderate cases, suggesting that co-infection with multiple pathogens may lead to worse clinical outcomes (*P* < 0.0001) (Supplementary Table 1). Notably, the occurrence of thrombotic events also significantly different between three groups (*P* < 0.0001).

Viral RNA was determined in time-interval nasopharyngeal swab samples obtained 7–42 dps, indicating that the rate of transition to negative viral RNA detection (CT value ≥ 40) gradually decreased with increasing disease severity among three patient groups (Fig. 1d). Among them, 34% of moderate patients achieved virus undetectable status at 7 dps (Fig. 1d, left panel), severe patients occurred between 7–14 dps (Fig. 1d, middle panel), while critically ill patients experienced a slow transition to virus undetectable between 21–28 dps (Fig. 1d, right panel). Meanwhile, a comparison of the upper respiratory tract viral load (Fig. 1e) and the viral kinetics fitting model (Fig. 1f) further confirmed above observation that a significant elevation in viral load (Fig. 1e) and a gradual decrease in the viral clearance (Fig. 1f) with increasing COVID-19 severity (reflected by gradual slope) particularly during the early (7–14 dps) and middle stages (21–28 dps) of acute infection.

### Early and robust virus-specific CD4^+^ and CD8^+^ T cell responses are crucial immune effectors for COVID-19 disease outcome

**Fig. 2:**
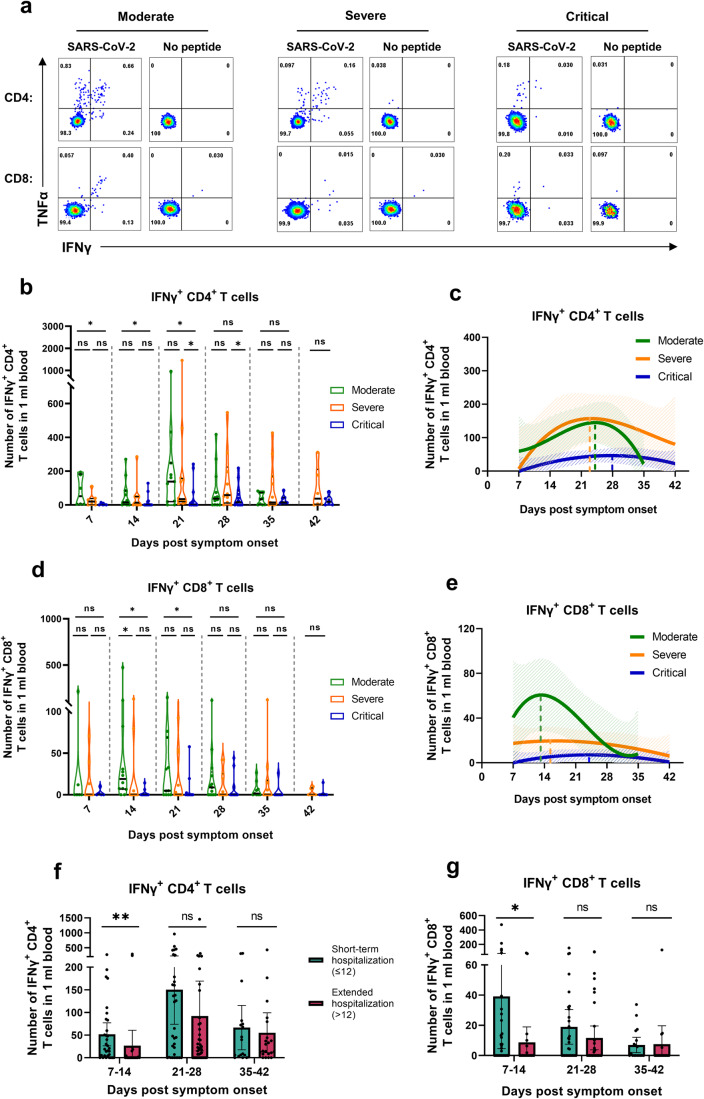
Virus-specific T cell response following acute infection across COVID-19 severity. **a** Representative flow cytometry plots showing virus-specific (IFNγ^+^) CD4^+^ and CD8^+^ T cell responses during the acute infection period in the moderate (23 dps), severe (21 dps), and critical (26 dps) groups. **b**, **d** Longitudinal analysis of absolute counts of virus-specific CD4^+^ (**b**) and CD8^+^ (**d**) T cells in peripheral blood (PB) samples per mL in the three groups (moderate, 7 dps *n* = 6, 14 dps *n* = 13, 21 dps *n* = 11, 28 dps *n* = 13, 35 dps *n* = 9; severe, 7 dps *n* = 8, 14 dps *n* = 9, 21 dps *n* = 12, 28 dps *n* = 11, 35 dps *n* = 11, 42 dps *n* = 6 ; critical, 7 dps *n* = 5, 14 dps *n* = 13, 21 dps *n* = 13, 28 dps *n* = 12, 35 dps *n* = 7, 42 dps *n* = 6). The black lines within the violin plots represent median and percentiles. **c**, **e** Kinetics of virus-specific CD4^+^ (**c**) and CD8^+^ (**e**) T cell responses in the moderate, severe, and critical groups following acute infection, using a polynomial nonlinear regression model with 95% confidence intervals visually represented by the shaded colors. The peak points are marked with colored dashed lines. **f**, **g** Comparison of virus-specific CD4^+^ (**f**) and CD8^+^ (**g**) T cell responses between the short-term (length of stay ≤ 12 days) and extended hospitalization groups (length of stay >12 days) (short-term, 7–14 dps n = 31, 21–28 dps *n* = 32, 35–42 dps *n* = 18; extended, 7–14 dps *n* = 22, 21–28 dps *n* = 39, 35–42 dps *n* = 21). Each dot represents one donor. Comparisons were performed using Mann–Whitney tests. *P < 0.05, **P < 0.01, ***P < 0.001

To profile the initial SARS-CoV-2-specific cellular immune response in relation to COVID-19 severity and clinical outcome, we measured the number of virus-specific (IFNγ^+^) T cells in acute phase (Supplementary Fig. 1a). The results (Fig. 2a, b) showed that patients with moderate disease who cleared the virus early and recovered rapidly had the highest number of virus-specific CD4^+^ T cells in the early (7–14 dps) and middle stages (14–21 dps) post-infection, significantly higher than critically ill patients (*P* < 0.05), and also trendily higher than that of severe patients. Notably, the IFNγ^+^CD4^+^ T cell responses of the patients with severe disease progressively increased (Fig. 2b), reaching a comparable level to the moderate group at approximately 21 dps, and remained at highest level among the three groups at late phase (28–42 dps). The kinetics analysis of virus-specific CD4^+^ T cells (Fig. 2c) exhibited that moderate and severe cases have similar peak times, which occurred around the third-week post-infection (21–22 dps), similar to previous report,^6^ and remarkably earlier than that observed in critically ill patients at 28 dps (Supplementary Table 2). Therefore, the delayed and insufficient virus-specific CD4^+^ T cell response may correlate with the worse clinical outcome in critically ill patients.

An early and robust IFNγ^+^CD8^+^ T cell response was also observed in moderate cases, significantly higher than that in severe (*P* < 0.05) and critical groups (*P* < 0.05) (Fig. 2d). This strong cytotoxic T lymphocyte (CTL) response peaked at around 13 dps (Fig. 2e) and then declined rapidly over time, potentially correlating with early disease control. This observation is similar to our previous finding in influenza virus H7N9, wherein early robust virus-specific CD8^+^ T cells contributed to the control of disease progression.^18^ A suboptimal virus-specific CD8^+^ T cell response characterized by a weaker magnitude and slightly delayed peaking at 17 dps (Fig. 2d, e), manifested in severe COVID-19 cases (Supplementary Table 2). In contrast, critically ill patients exhibited the lowest SARS-CoV-2-specific CD8^+^ T cell response with remarkably delayed production kinetics, which further impaired early viral clearance and leading to the poorest clinical outcomes (Fig. 2d, e). Furthermore, to explore the relationship between the virus-specific T cell responses and hospitalization duration, we divided the patient cohort into two groups based on a cut-off of 12 days, the mean timepoint at which COVID-19 recovery and discharge observed in the present study. We found that patients with shorter hospitalization have more robust of both IFNγ^+^CD4^+^ and CD8^+^ T cell responses than those with extended hospitalization (*P* < 0.05) during the early phase post-infection (7–14 dps) (Fig. 2f, g). Taken together, the above data suggest that early robust virus-specific CD4^+^ and CD8^+^ T cell responses control COVID-19 severity, whereas delayed and low-level antiviral adaptive immune responses, especially CD8^+^ T cells, are associated with worse clinical progression.

The innate immune responses, including the absolute numbers of natural killer (NK) and NKT cell populations (Supplementary Fig. 1a), were also evaluated and showed significantly decreased in response levels with increasing disease severity (Supplementary Fig. 2a, b). These differences gradually diminished and reached similar levels in three groups at 35–42 dps of late-stage post infection. This finding suggests that the innate immune response assists in the response against early SARS-CoV-2 infection, and the decreased number of NK and NKT cells in the blood is related to the COVID-19 severity, which may be the collaborative result of severe lymphocyte suppression (Supplementary Fig. 2c–f) and lung infiltration.^19^

### Delayed antiviral antibody production is associated with severe and critical COVID-19

**Fig. 3:**
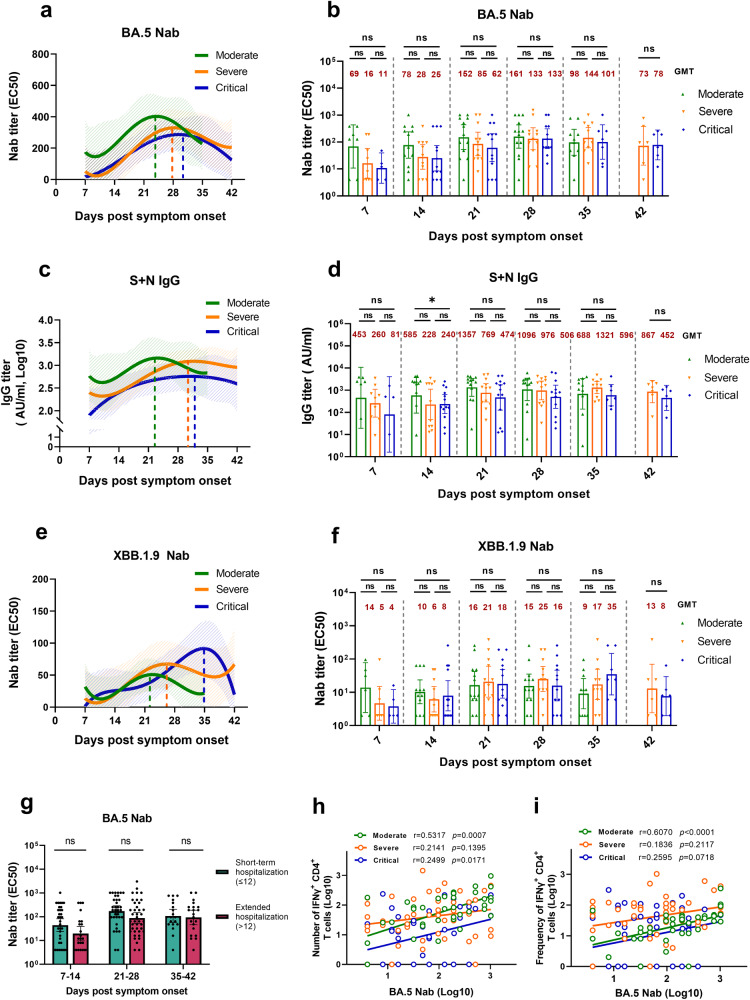
Acute antivirus antibody responses across COVID-19 severity. **a**, **c**, **e** Kinetics of serum BA.5 Nab (**a**), anti-S + N IgG (**c**), and XBB.1.9 Nab (**e**) in the moderate, severe and critical groups following acute infection, using a polynomial nonlinear regression model with 95% confidence intervals visually represented by the shaded colors. The peak points are marked with colored dashed lines. **b**, **d**, **f** Comparison of geometric mean titers (GMTs) of anti-viral antibodies in longitudinal serum samples, including BA.5 Nab (**b**), anti-S + N IgG (**d**), and XBB.1.9 Nab (**f**) in the moderate, severe, and critical groups (moderate, 7 dps *n* = 7, 14 dps *n* = 12, 21 dps *n* = 13, 28 dps *n* = 13, 35 dps *n* = 10; severe, 7 dps *n* = 9, 14 dps *n* = 10, 21 dps *n* = 12, 28 dps *n* = 11, 35 dps *n* = 11, 42 dps *n* = 7; critical, 7 dps *n* = 5, 14 dps *n* = 13, 21 dps *n* = 13, 28 dps *n* = 12, 35 dps *n* = 7, 42 dps *n* = 6). Titers presented by GMT with 95%CI. **g** Comparison of BA.5 Nab GMT between the short-term (length of stay ≤ 12 days) and extended hospitalization groups (length of stay > 12 days) (short-term, 7–14 dps *n* = 33, 21–28 dps *n* = 34, 35–42 dps *n* = 20; extended, 7–14 dps *n* = 23, 21–28 dps *n* = 40, 35–42 dps *n* = 21). Titers presented by GMT with 95%CI. **h**, **i** Correlation between BA.5 Nab (Log10) and the number (**h**) or frequency (**i**) of IFNγ^+^CD4^+^ T cells ^(^Log10) in the moderate, severe, and critical groups. Frequency values are first multiplied by 1000 and then Log10 transformed. Spearman’s correlation coefficients and significance are shown accordingly. Each dot represents one donor. Comparisons were performed using Mann–Whitney tests. *P < 0.05, **P < 0.01, ***P < 0.001

To investigate whether and how antibody-mediated humoral immune responses differ across COVID-19 severity, we assessed the SARS-CoV-2-specific antibody titers and kinetics in time-interval plasma samples during acute infection. The Nab kinetic fitting model revealed that a progressive delay in the peaking time of BA.5 Nab (Fig. 3a) occurred from moderate (peak time, 24 dps), severe (peak time, 28 dps) to critical ill groups (peak time, 30 dps), suggesting that the antiviral Nab production capacity is limited and delayed by clinical severity (Supplementary Table 2). More antibody types, including the binding antibody anti-S + N IgG (Fig. 3c) and Nab against XBB.1.9 (Fig. 3e), were further assayed, demonstrating similar delayed generation trends (Supplementary Table 2). Notably, no statistical differences in the geometric mean titers (GMTs) of BA.5 Nab were observed among the three groups (Fig. 3b), although trendily higher titer was observed in patients with moderate disease at 7–28 dps. At the late infection stage (35–42 dps), BA.5 Nab titers in the severe and critical groups gradually surpassed those in the moderate group, similar to other COVID-19 cohorts,^5,20^ indicating a potential association with faster antibody decay in the patients with moderate disease after recovery and sustained antibody generation in severely infected cases due to longer and higher antigen exposure. In addition, the analysis of GMTs of XBB.1.9 Nab and anti-S + N IgG revealed similar findings (Fig. 3d, f). Even after further grouping and comparison based on hospitalization duration, no significant differences were observed in the GMT of BA.5 Nab between short- and long-term hospitalized patients (Fig. 3g). Moreover, when comparing anti-BA.5 Nab and IFNγ^+^ T cell responses between vaccinated and unvaccinated critical patients (Supplementary Table 3), we did not find evidence that the vaccination effectively influenced the levels and kinetics of above adaptive immunity (Supplementary Fig. 3a–c). Collectively, these data suggest that delayed antiviral antibody production rather than their levels correlate with disease severity.

To determine whether the correlation exists between virus-specific cellular and humoral immunity responses^21,22^ in the context of the varying acute COVID-19 severities. The frequency and absolute number of IFNγ^+^CD4^+^ and CD8^+^ T cells were analyzed for correlations with BA.5 Nab level in the three groups. Unexpectedly, our data demonstrated that patients with moderate disease exhibited a positive correlation between the frequency (*r* = 0.6070, *P* < 0.0001) and absolute count (*r* = 0.5317, *P* = 0.0007) of SARS-CoV-2-specific CD4^+^ T cells and BA.5 Nab titers (Fig. 3h, i). However, no such correlation was observed in the severely and critically ill groups. Meanwhile, no apparent correlation between the frequency or number of virus-specific CD8^+^ T cells and BA.5 Nab titers among the three groups of patients was observed (Supplementary Fig. 3d–g).

### Slow decay and sustained maintenance of SARS-CoV-2-specific antibodies in individuals recovered from severe COVID-19

**Fig. 4:**
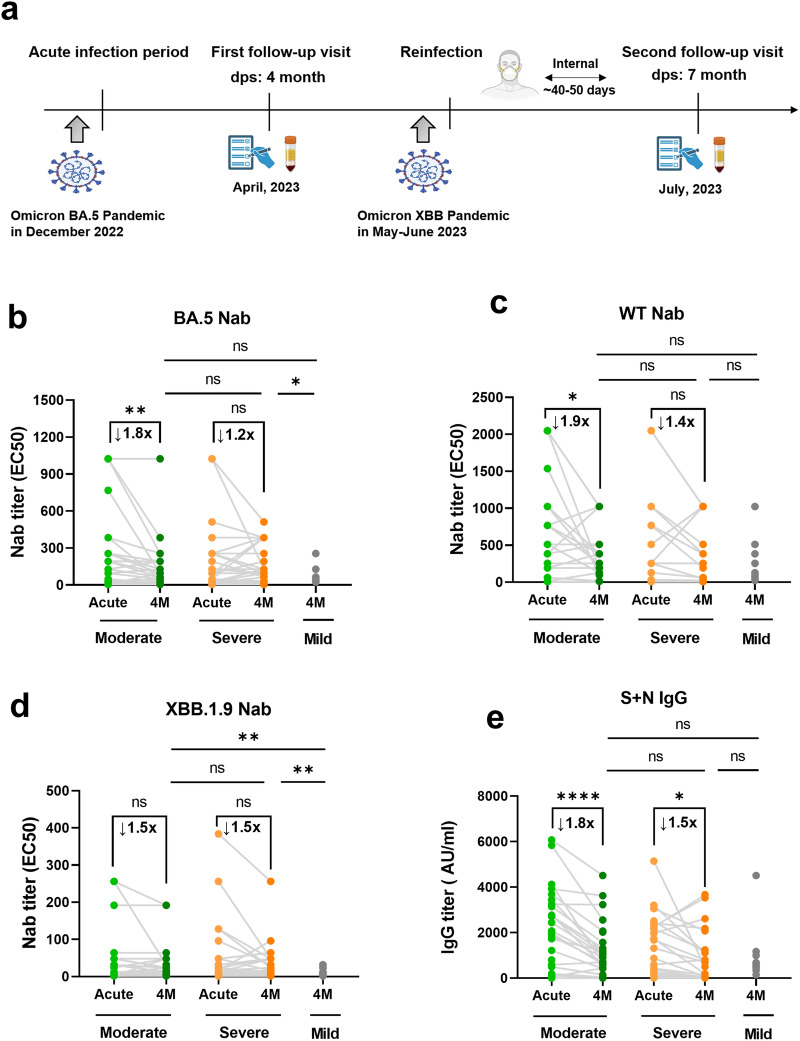
Decay and maintenance of antiviral antibodies in the COVID-19 recovery individuals. **a** Timeline diagram of the different SARS-CoV-2 subvariants pandemic in China and follow-up of the COVID-19 convalescent cohort. **b–e** Comparison of paired plasma antibody titers from the acute infection period to 4 months after recovery, including BA.5 Nab (**b**), WT Nab (**c**), XBB.1.9 Nab (**d**), and anti-S + N IgG (**e**) in the mild (*n* = 13), moderate (*n* = 25) and severe groups (*n* = 24). Each dot represents one donor. Comparisons for paired samples were performed using the Wilcoxon matched-pairs signed rank test, with the fold decline marked in black font at the top. Comparisons between groups were performed using Mann–Whitney tests. *P < 0.05, **P < 0.01, ***P < 0.001

Follow-up visits were conducted at the 4-month (4 M) and 7-month (7 M) following acute Omicron BA.5 infection to investigate the influence of the initial disease severity on the formation and maintenance of adaptive immunity memory (Fig. 4a). No participant in our cohort reported SARS-CoV-2 PCR^+^ test or symptomatic reinfection at the 4 M visit (Supplementary Table 4), which is consistent with the epidemiological data published by the Chinese Center for Disease Control and Prevention (CCDC) (https://www.chinacdc.cn/).

Our acute-4M paired data revealed that the degree of viral antibody decay and maintenance capacity differed across the initial COVID-19 severities (Fig. 4b–e). Specifically, significant decreases in wild-type (WT) Nab (1.9-fold, *P* = 0.0154) and BA.5 Nab (1.8-fold, *P* = 0.0055) titers were observed in patients with initial moderate disease (Fig. 4b, c). However, only minimal declines were observed in severe patients for both BA.5 Nab (1.2-fold, *P* = 0.76) and WT Nab (1.4-fold, *P* = 0.087) titers. In contrast to Nabs, binding antibody anti-S + N IgG appeared to decay faster and showed a significant titer decrease in both the moderate and severe groups (1.8-fold, *P* < 0.0001 for Moderate; 1.5-fold, *P* = 0.015 for Severe), suggesting that different antibody types have distinct temporal characteristics due to their divergent antiviral mechanisms.

Due to the high mortality and loss of follow-up among critically ill hospitalized patients, mild convalescents were supplemented as controls. Subsequently, the cross-sectional antiviral antibody titers at 4 M for individuals who had recovered from different initial severities were compared. Consistent with data on antibody decay, we found that individuals who recovered from severe infection had the highest levels of BA.5 and XBB.1.9 Nabs at 4 M, significantly higher than those of mild convalescents (*P* < 0.05) and trendily higher than moderate convalescents (Fig. 4b, d). The XBB.1.9 Nab levels of the moderate were also higher than those recovered from mild disease (*P* < 0.05, Fig. 4d), whereas the levels of other antibody types were similar. In conclusion, we found that patients with severe COVID-19 who underwent prolonged hospitalization and higher antigen exposure exhibited the slowest antibody decay and most sustained antibody maintenance during convalescence.

### Initial COVID-19 severity imprints T-cell memory maintenance and influences their differentiation

**Fig. 5:**
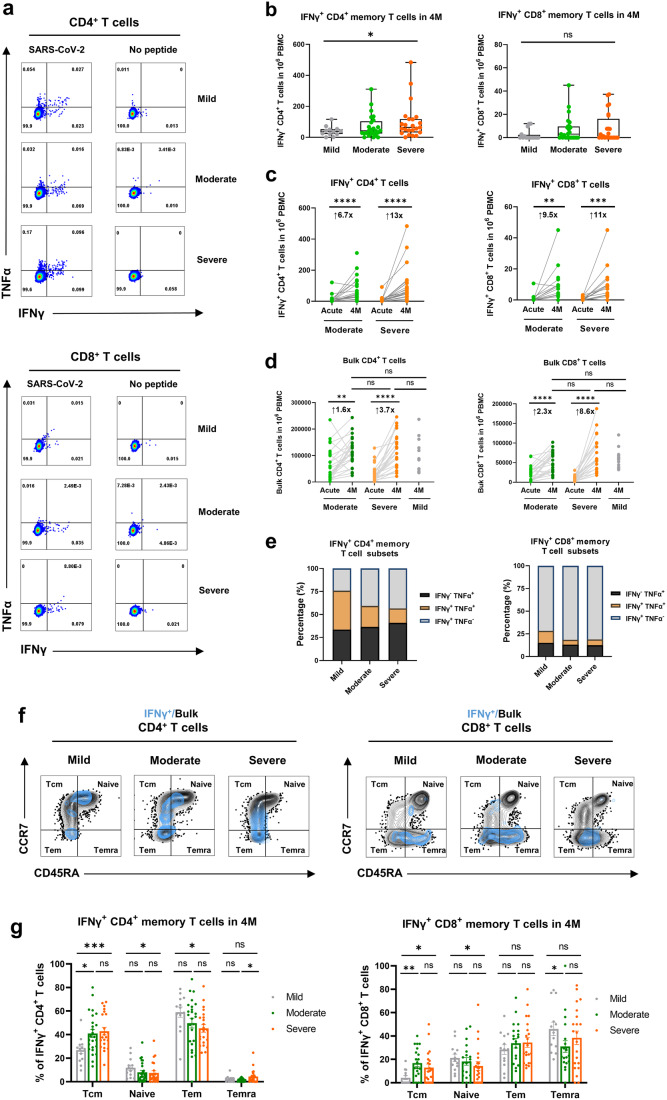
Memory T cell maintenance and differentiation across initial COVID-19 severity. **a** Representative flow cytometry plots showing virus-specific CD4^+^ and CD8^+^ T cell responses (IFNγ^+^) at 4 months after COVID-19 recovery in the mild, moderate and severe groups. **b** Comparison of the number of virus-specific memory CD4^+^ and CD8^+^ T cell responses in 4 M between individuals recovered from initial mild (*n* = 13), moderate (*n* = 25) and severe (*n* = 24) infection. The black lines within the box plots represent median. **c** Comparison of the number change of IFNγ^+^CD4^+^ and CD8^+^ T cells from the acute phase to 4 M in the moderate (*n* = 25) and severe (*n* = 24) groups. **d** Comparison of the numbers of bulk CD4^+^ and CD8^+^ T cells from the acute phase to 4 M in the moderate (*n* = 25), severe (*n* = 24) and mild (*n* = 13) groups. **e** Analysis of the multi-functionality of virus-specific memory CD4^+^ and CD8^+^ T cells in the mild, moderate, and severe groups at 4 M. **f** Representative overlay flow cytometry plots showing the bulk (black) and virus-specific (blue) CD4^+^ and CD8^+^ memory T cell phenotypes divided by CD45RA and CCR7 in the mild, moderate, and severe groups. **g** Frequency of different virus-specific memory T cell phenotypes in the mild (*n* = 13), moderate (*n* = 25) and severe (*n* = 24) groups in 4 M. Each dot represents one patient. Comparisons of paired samples were performed using the Wilcoxon matched-pairs signed rank test, with fold increase marked in black font at the top. Comparisons between groups were performed using Mann–Whitney tests. *P < 0.05, **P < 0.01, ***P < 0.001

Further assessment and analysis of the SARS-CoV-2-specific T-cell memory at 4 M revealed the enhanced IFNγ^+^CD4^+^ and CD8^+^ memory T cell responses (Supplementary Fig. 1b) with increasing in the initial COVID-19 severity from mild to moderate to severe (Fig. 5a, b). Specifically, severe convalescents exhibited the highest virus-specific CD4^+^ memory T cell response (Fig. 5b, left), which was significantly (and markedly) higher than those in the mild-recovery (*P* < 0.05) and suboptimal moderate groups (*P* = 0.179). The number of virus-specific CD8^+^ T memory cells (Fig. 5b, right) showed a similar yet insignificantly increasing trend across initial disease severity among the three groups. Notably, the analysis of paired peripheral blood mononuclear cells (PBMCs) between acute phase and 4 M, revealed a more significant increase in both the number (Fig. 5c) and frequency (Supplementary Fig. 4a, b) of IFNγ^+^CD4^+^ and CD8^+^ T cell responses in severe individuals compared to moderate individuals. Importantly, we found that lymphocyte suppression or depletion in hospitalized patients with acute SARS-CoV-2 infection (Supplementary Fig. 2c–f) was completely restored at 4 M, and the bulk CD4^+^ and CD8^+^ T cell responses were comparable between mild, moderate and severe convalescents (Fig. 5d). Moreover, functional cytokine analysis revealed that compared with the convalescents in the moderate and severe groups who underwent hospitalization, the individuals in the mild group exhibited stronger multi-functionality (TNFα^+^IFNγ^+^) in SARS-CoV-2-specific CD4^+^ and CD8^+^ memory T cells (Fig. 5e).

To investigate the differentiation phenotypes of SARS-CoV-2-specific memory T cell subsets in 4 M post-recovery among the three groups (Fig. 5f), memory subpopulations were categorized into central memory T cells (Tcm, CD45RA^–^ CCR7^+^), effector memory T cells (Tem, CD45RA^–^ CCR7^–^), terminally differentiated effector cells (Temra, CD45RA^+^ CCR7^–^), and naïve T cells (Tnaive, CD45RA^+^ CCR7^+^). Our data demonstrated that the virus-specific CD4^+^ memory T cell subset was predominantly comprised Tcm and Tem, whereas the virus-specific CD8^+^ memory T cell population was primarily composed of Temra and Tem (Fig. 5g and Supplementary Fig. 4c, d), consistent with the finding of previous reports.^12,23,24^ However, the phenotypic features of virus-specific memory T cells differed according to the initial severity, with moderate and severe convalescents who underwent hospitalization exhibiting a higher Tcm and lower naïve phenotype in virus-specific memory CD4^+^ and CD8^+^ T cell subsets. In contrast, individuals with initial mild infection exhibited higher naïve and Tem or Temra cells in the virus-specific memory CD4^+^ and CD8^+^ T cell subsets (Fig. 5g).

### SARS-CoV-2 reinfection risk inversely correlates with initial COVID-19 severity

**Fig. 6:**
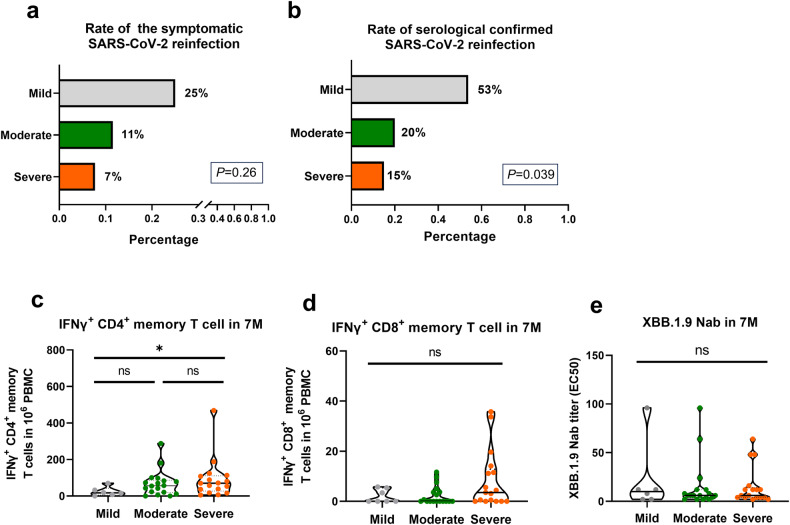
Subsequent reinfection risk and long-term immune maintenance among individuals with distinct initial COVID-19 severity. **a**, **b** Analysis of the symptomatic SARS-CoV-2 reinfection rate (**a**) and the serologically confirmed (**b**) reinfection rate (4-fold increase in XBB.1.9 Nab between 4M and 7M) in the mild, moderate and severe convalescent groups at 7 months post acute infection. **c**–**e** Comparison of the number of virus-specific memory CD4^+^ (**c**) and CD8^+^ (**d**) T cell responses and XBB.1.9 Nab titers (**e**) between non-reinfected individual of mild (*n* = 6), moderate (*n* = 18) and severe (*n* = 17) groups in 7 M. The black lines within the violin plots represent median and percentiles. Reinfection risk between groups were assessed using chi-square test. Comparisons for paired samples were performed using the Wilcoxon matched-pairs signed rank test, with fold increase marked in black font at the top. Comparisons between groups were performed using Mann–Whitney tests. *P < 0.05, **P < 0.01, ***P < 0.001

Since initial COVID-19 severity influences the formation and differentiation of long-term immune memory (Figs. 4, 5), we subsequently addressed whether initial severity influences reinfection risk in subsequent SARS-CoV-2 variant waves. The second follow-up visit was conducted after the second SARS-CoV-2 wave of XBB variant pandemic in China (Fig. 4a). A total of 13% participants (9/68) reported symptoms associated with acute upper respiratory tract infection and tested positive for SARS-CoV-2 antigen or PCR (Supplementary Table 4). Consequently, 25% of mild participants (4/16), 11% of moderate participants (3/26), and 7% of severe participants (2/26) were identified as symptomatic SARS-CoV-2 reinfections (Fig. 6a). Furthermore, by quantifying the XBB.1.9 Nab in paired plasma samples (4M-7M), a four-fold increase in titers was used as the criterion to confirm reinfection in individuals. Based on this standard, serological confirmed reinfection rates differed significantly (*P* = 0.036) among the three groups with 53% of mild participants (6/13), 20% of moderate participants (4/20), and 15% of severe participants (3/19) (Fig. 6b). Therefore, our serological results supported that initial severity did influence the subsequent reinfection risk, as reported using a questionnaire (Fig. 6a).

We compared the number and percentage of virus-specific CD4^+^ and CD8^+^ memory T cells between individuals with and without subsequent reinfection in 4 M (Supplementary Fig. 5a–d). Although non-reinfected individuals exhibited a slightly higher IFNγ^+^CD4^+^ and CD8^+^ T cell responses at 4 M post BA.5-infection, no statistically significant differences were found. More importantly, the analysis of the virus-specific immune effectors in the 7 M (Fig. 6c–e) among non-reinfected individuals revealed that even though the XBB Nab titers reduced to closely comparable levels across the groups, individuals who had recovered from severe COVID-19 still exhibited significantly higher IFNγ^+^CD4^+^ T cell responses (*P* < 0.05) and trendily higher IFNγ^+^CD8^+^ T cell responses (*P* = 0.196) than mild convalescents (Supplementary Table 5). The memory phenotypes of IFNγ^+^CD4^+^ and CD8^+^ memory T cells at 7 M within non-reinfected individuals were similar to those at 4 M (Supplementary Fig. 5e, f). Additionally, despite the potential limitations in statistical analysis due to a small number of reinfected individuals in each group (mild, *n* = 6; moderate, *n* = 4; severe, *n* = 3), our data suggest that the increase level of adaptive immune response recall after reinfection also appears to be inversely influenced by the initial severity (Supplementary Fig. 5g–i). Notably, increased initial COVID-19 severity may enhance individuals’ self-protection, potentially impacting the reinfection risk to some extent. However, under longer observation periods post initial infection ( > 6 months) and in the context of national easing of epidemic prevention and control, this influence may be relatively minimal. Taken together, our data demonstrated that greater initial COVID-19 severity reduces the subsequent reinfection risk, and which may associate with the sustained maintenance of the virus-specific memory T cell responses.

## Discussion

**Fig. 7:**
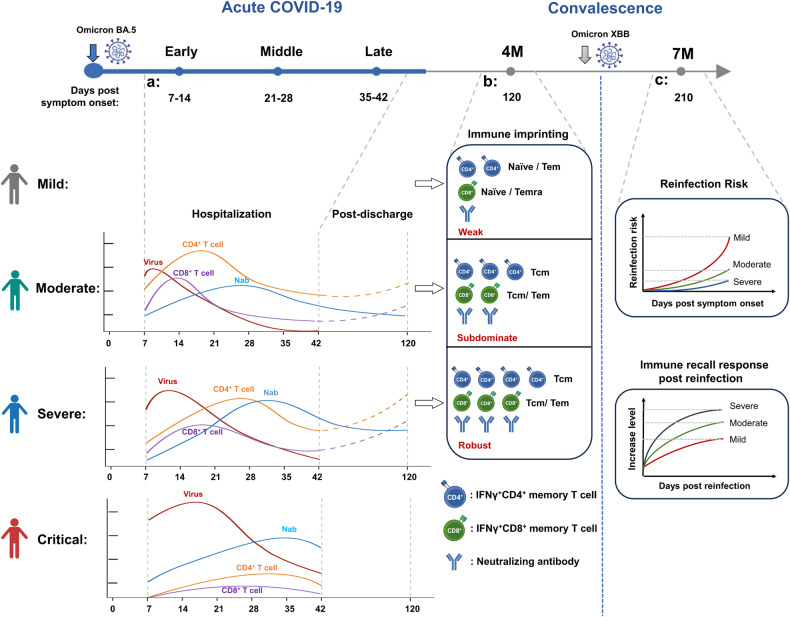
Schematic representation of acute and long-term immune effectors kinetics, and reinfection risk, across initial COVID-19 severity. **a** Kinetics representation of virus-specific immune effectors and virus loads in relation to clinical severity during acute SARS-CoV-2 infection. **b** The profile of virus-specific T-cell memory response and neutralizing antibody maintenance across initial COVID-19 severity in 4-month post infection. **c** The reinfection risk and immune recall response post reinfection in 7-month post initial COVID-19 across severity

The relationship between acute virus-specific T cell response and COVID-19 disease severity, and the influence of disease severity on T memory formation and differentiation has always been investigated by cross-sectional studies.^3,10,11,25,26^ These either focus on the acute infection phase to study the T cell response and disease outcome or aim to compare the magnitude of memory T cell response during convalescence. Moreover, few studies have investigated the effects of the initial severity of preceding SARS-CoV-2 infection on the reinfection risk, probably because such study need long-term observation. In the present study, using a systematically longitudinal COVID-19 cohort, we filled the knowledge gaps in relationship between the early and long-term virus-specific adaptive immune responses and initial COVID-19 severity, and their subsequent influence on the reinfection risk (Fig. 7).

Lucus et al. reported that COVID-19 severity correlates with the delayed Nabs production kinetics but does not correlate with the cross-sectional antiviral antibody levels.^5^ Moderbacher et al. argued that the virus-specific T cells based on OX40^+^CD154^+^CD4^+^ or AIM^+^CD69^+^CD8^+^ and Nabs need to coordinate together to limit the disease severity, and that Nab titers are not predictive of reduced disease severity.^3^ Our previous study indicated that T cell response measured by CD38^+^HLADR^+^ CD4^+^ and CD8^+^ drives the recovery of severe COVID-19 when Nabs were generated to a certain level.^4^ In current study, our data supports and extends these findings in a larger cohort with a more detailed categorization of disease, and from different virus-specific T cell assay perspective, employing IFNγ^+^ T cells.^27^ Above independent studies summarized and pinpointed a conclusion that virus-specific CD4^+^ and CD8^+^ T cell responses, instead of Nabs, drive COVID-19 recovery, but a certain level of Nabs (serological response threshold) must exist.

The causes underlying the delay and impairment of the acute virus-specific immune effectors in patients with severe and critical COVID-19 are poorly understood. Previous studies^28,29^ revealed that aging and multiple comorbidities suppress early T cell responses and are associated with immunological aging. The present study also confirmed that age, gender, and coexisting disorders correlated with disease severity. Other immunological mechanisms may also be involved, including severe lymphopenia and apoptosis,^29^ abnormal chemokines production leading to lymphocyte redistribution,^19,29^ and the delayed virus-specific cTfh cells appearance resulting in delayed Nab generation.^20^

Severe/critical versus mild/moderate illness for sure provide differential T cell activation signal: different antigen dose, different antigen exposure time,^30^ and different cytokines environment.^19^ How these factors influence the later formation of T cell memory? In our study, mild/moderate cases generated more robust virus-specific T cell response with higher multi-functionality early during acute infection than those of severe groups, while severe COVID-19 cases induced higher and sustained virus-specific T cell response in later timepoints. Theoretically, this raised an evolutionary question of whether high-quality and strong-protective memory T cells are generated from early robust and multifunctional T cells in mild/moderated cases, or from high-dose and long-term antigen-experienced T cells in severe patients. This question has long been disputed,^31–34^ with one hypothesis arguing for the multifunctional memory T cells that secrete various cytokines, whereas another suggest the phenotype-biased (mainly Tcm) memory T cells induced by prolonged antigenic stimulations, or a combination of both, but the influence of proportion should be taken into consideration. The present study provided a unique insight into this disputation and revealed that increased severity leads to a higher number of memory T cells, as corroborated by our phenotype data, which demonstrated that most virus-specific memory T cells from severe cases evolved quicker into central memory T cells rather than the more naïve and effector memory T cells in mild cases. More importantly, in addition to the phenotype experiments, the assessment of reinfection risk in the three groups provided real-world protection level evidence indicating that the memory immunity of severe patients is superior to that of mild and moderate patients. Our data on the fold increase in XBB Nab titers from 4 M to 7 M (before and after reinfection) further suggested that the quality of memory B cells recalled in the severe group was higher than that in the mild and moderate groups. These findings confirm and expand previous report that prior COVID-19 can provide protection against SARS-CoV-2 reinfection,^35^ and further indicate that this protection is influenced by initial disease severity.

Although the limited number of patient samples at certain acute-phase timepoints and follow-up participants, may have negatively impacted the group size balances and statistical analyses, and potential demographic confounders, such as age and general health status were neglected. Our longitudinal study systematically examined adaptive immunity in the acute and memory phases and real-world reinfection protection against SARS-CoV-2. We found that the strength and temporal characteristics of virus-specific T cells affect COVID-19 severity during the acute infection phase, and initial COVID-19 severity inversely imprints T-cell memory maintenance and stratifies reinfection risk in the subsequent waves of emerging variants (Fig. 7). The present study revealed a logical cycle between infection immunity and disease severity, similar to the concept of a Karma cycle in philosophy: when virus infection is severe (considered as bad), it leads to the generation of better memory adaptive immunity, thereby provides better protection against future infections (turning the initial bad outcome into a good one). Moreover, our reinfection risk assessment data also provided a useful insight for the management of future infections.

## Method

### Ethics statement

This study was approved by the Medical Ethics Committee of the First People’s Hospital of Yunnan Province (protocol ID: KHLL2023-KY098), and performed in accordance with the Declaration of Helsinki. All patients and volunteers provided written informed consent. The denoted primary contact of intensive care unit (ICU)-sedated patients was contacted to obtain initial oral and subsequently written consent.

### Study cohort

Acute COVID-19 patients confirmed by SARS-CoV-2 polymerase chain reaction (PCR) test were enrolled at First People’s Hospital of Yunnan Province in China between December 17, 2022 and May 1, 2023. Frequent longitudinal throat swabs and peripheral blood samples were obtained after hospital admission at time-intervals in 7–42 days, and each individual in acute phase provided 2–8 samples. Subsequently, we converted the sample collection time to days post symptom onset (dps), with a total of 169 throat swabs and 173 blood samples analyzed at 7-day intervals from 7 to 42 dps. Subsequently, individuals who had recovered from SARS-CoV-2 infection of varying initial severity were recruited to the follow-up cohort. Convalescents with previous mild infection were included as controls owing to the high rates of mortality and loss to follow-up among critically ill patients. Blood samples were collected from the follow-up participants during the acute phase and at 4 and 7 months. However, most patients with mild infection were not hospitalized during the acute phase of infection; consequently, the acute phase paired specimens were lacking for these patients.

Disease severity was assessed daily by reviewing electronic health records by a clinical respiratory physician, and patients were grouped according to the highest severity throughout the entire hospitalization period, based on the Diagnosis and Treatment Protocol for Novel Coronavirus Pneumonia (Trial Version 10) published by National Health Commission of China.^36^ Mild disease status characterized by the presence of only symptoms of upper respiratory tract infection such as sore throat, cough, and fever. Moderate disease status was defined as: (1) hospitalization, (2) characteristic “COVID-19 pneumonia” observed during imaging examination, and (3) a respiratory rate of < 30 times/min and oxygen saturation is > 93% when breathing air in the resting state. Severe disease status was characterized by the presence of the following features: (1) progressive exacerbation of clinical symptoms, and imaging examinations exhibiting an increase of > 50% in pulmonary lesions within 24–48 h; (2) arterial partial pressure of oxygen (PaO2)/fraction of inspired oxygen (FiO2) of ≤ 300 mmHg (1 mmHg = 0.133 kPa); and (3) respiratory rate ≥ 30 times/min or oxygen saturation ≤ 93%. Critical disease status was characterized by the presence of the any of following conditions: (1) respiratory failure necessitating mechanical ventilation; (2) shock, and (3) multiple organ failure necessitating ICU monitoring and treatment. For every patient, the time since the onset of symptoms was determined in the following prioritized manner: (1) the explicit earliest symptom onset dates reported by patients; (2) physician estimated dates through careful evaluation of the electronic health records and positive PCR test time.

### Viral RNA measurements

Longitudinal nasopharyngeal swab samples were collected approximately every 3–7 days during the acute infection course (7–42dps). The viral RNAs were detected using commercial kits (Sansure Biotech, Changsha, China) targeting the ORF1ab and N genes based on quantitative reverse transcription polymerase chain reaction (qRT-PCR) approved by the China National Medical Products Administration (NMPA). The specimens were considered positive if the Ct value was < 40, and negative if the results were Ct value ≥ 40 or undetermined.

### SARS-CoV-2 conventional virus neutralization test

Plasma samples were collected by centrifuging whole blood at 800 × g for 10 min at 25 °C. The neutralizing activity of plasma was evaluated by utilizing a cytopathic effect (CPE)-based assay in a certified BSL-3 laboratory, as described previously.^27^ Fifty microliters of plasma were two-fold serially diluted, mixed with 100 TCID50 SARS-CoV-2 Wuhan-1 (WT), Omicron BA.5 or XBB 1.9 viral solution in 96-well flat-bottom plates, and incubated for 2 h at 37 °C and 5% CO_2_. Then, 1.2 × 10^4^ VERO E6 cells were seeded in the virus–plasma mixtures. After incubation (37 °C, 4 days), CPE was evaluated utilizing a Celigo Imaging Cytometer (Nexcelom Bioscience, Lawrence, MA, USA). The presence or absence of CPE was determined by comparing to positive and negative control plasma sample, and the final neutralizing antibody titer of each sample was calculated from two replicate wells.

### iFlash-SARS-CoV-2 IgG assay

The detection of anti-S + N IgG antibodies against SARS-CoV-2 were performed by iFlash Immunoassay Analyzer in accordance with the manufacturer’s instructions (Shenzhen Yhlo Biotech, Shenzhen, China), as described previously.^37^ One hundred microliters of plasma sample were incubated with SARS-CoV-2 antigen-coated paramagnetic microparticles to allow the antibody binding. After the addition of an acridinium-labelled anti-human IgG conjugate and trigger solutions to the reaction mixture, the iFlash optical system was used to measure the anti-S + N IgG titre.

### SARS-CoV-2 peptide pool

As previously described,^27,38^ four hundred and forty-seven 15-mer peptides (overlapping by 11 amino acids) spanning the entire SARS-CoV-2 structural protein region of spike (S), membrane (M), nucleocapsid (N), and envelope (E) were designed and generated by the PEPTIDE 2.0 online generator (https://www.peptide2.com/) and synthesized by GL Biochem Corporation (Shanghai). Each peptide had a purity over 80% and was dissolved in DMSO, and then pooled with each at a concentration of 45 μM to create a stock solution.

### PBMC isolation and ex vivo stimulation

PBMCs were isolated from heparinized whole blood by density-gradient sedimentation using Ficoll-Paque according to the manufacturer’s instructions (GE Healthcare, Cat#17–1440–02). The utilization of virus peptide pool for PBMCs stimulation has been previously established.^27,38^ 5 × 10^5–1^ × 10^6^ PBMCs were then cultured in RPMI 1640 medium (Gibco) added with 10% heat-inactivated FBS (Biological Industries, Israel Beit-Haemek), 100 U/ml penicillin (Gibco) and 0.1 mg/ml streptomycin (Gibco). The PBMCs were treated with the SARS-CoV-2 peptide pool (250 nM/each peptide) and 20 U/mL recombinant interleukin-2 (rIL-2) and 1 μM GolgiPlug (BD Biosciences) overnight (16 h) at 37 °C and 5% CO_2_.

### Flow cytometry

In the flow cytometry panel for acute infection samples, 5 × 10^5^–1 × 10^6^ cells harvested from the overnight stimulation were washed and incubated with Live/Dead Aqua V510 (Thermo, Cat# L34957) for 20 min on ice. The surface-staining was then performed on ice for 30 min with the following antibodies: anti-CD3-PECF594 (BD, Cat# 562280, 1:200, clone UCHT1), anti-CD4-BV650 (Biolegend, Cat# 317435, 1:200, clone OKT4), anti-CD8-PerCP-Cy5.5 (BD, Cat#341051, 1:50, clone SK1), anti-CD14-APC-H7 (BD, Cat# 560180, 1:100, clone MφP9), anti-CD19-APC-H7 (BD, Cat# 560177, 1:100, clone SJ25C1) and anti-CD56-PE-Cy7 (BD, Cat# 335791, 1:100, clone NCAM16.2). After fixation and permeabilization with Cytofix and Perm (BD, Cat# 554714) on ice for 20 min, intracellular staining was performed on ice for 30 min with anti-IFN-γ-AF700 (BD, Cat# 557995, 1:200, clone B27), anti-TNF-α-APC (BD, Cat# 340534, 1:50, clone 6401.1111). Then, PBMCs were resuspended in 300 μL FACS buffer, and loaded to FACS Fortessa instrument (BD, Cat# Bioscience) to acquire data, and then analyzed using FlowJo software version 10.8.1 (Tree Star, Inc., Ashland, OR, USA).

The operating procedure for the flow cytometry panel for acute-convalescent paired samples is the same as the procedure described above. However, the following adjustments were made: 1 × 10^6^–1.5 × 10^6^ cells were harvested; dead cell discrimination marker Live/Dead-FVS440 (BD, Cat# 566332); surface-stained antibodies: anti-CD3-BUV395 (BD, Cat# 564001, 1:200, clone SK7), anti-CD4-APC-H7 (BD 560158, 1:150, clone RPA-T4), anti-CD8-PerCy5.5 (BioLegend, Cat# 344710, 1:200, clone SK1), anti-CCR7-APC (BioLegend, Cat# 353214, 1:150, clone G043H7), and anti-CD45RA-FITC (BioLegend, Cat# 304106, 1:200, clone HI100); intracellular staining antibodies: anti-IFN-γ-BV421 (BioLegend, Cat# 506538, 1:200, clone B27) and anti-TNF-α-PE (BD, Cat# 559321, 1:200, clone MAb11).

### Statistical analyses

All statistical analyses were performed using GraphPad Prism software version 9.5.1 and SPSS version 26. Differences between groups were considered statistically significant if *P* < 0.05. The two-tailed Mann‒Whitney test was conducted for non-normally distributed data (presented as the median and percentiles), and t-test was employed for normally distributed data (presented as the mean ± SEM). Antibody titers are presented as geometric mean titers (GMTs) with 95% confidence interval. The Wilcoxon rank-sum test was used to compare paired continuous variables not normally distributed. Analysis of virus clearance and immune parameters against days post symptom onset were examined by polynomial nonlinear regression with 95% confidence intervals. Correlation analysis was performed using Spearman and linear regression. Gender, comorbidity, clinical outcomes, and reinfection risk between groups were assessed using chi-square test.

### Supplementary information

Supplementary Materials

## Data Availability

All data supporting the findings of this research are available within the article and its supplementary information or from the corresponding author upon reasonable request.
