# Supplementary material for: Initial COVID-19 severity influenced by SARS-CoV-2-specific T cells imprints T-cell memory and inversely affects reinfection
Source: Signal Transduct Target Ther. 2024 May 29;9:141. doi: 10.1038/s41392-024-01867-4 (PMC11136975; doi:10.1038/s41392-024-01867-4)
Supplement: Supplementary file 1 — Supplementary Materials [file 41392_2024_1867_MOESM1_ESM.docx]

Supplementary Materials for

Initial COVID-19 severity influenced by SARS-CoV-2-specific T cells

imprints T-cell memory and inversely affects reinfection

Gang Yang^1,3#^, Jinpeng Cao^2,3#^, Jian Qin^1#^, Xinyue Mei^2,3#^, Shidong Deng^2,3^, Yingjiao Xia^1^,

Jun Zhao^1^, Junxiang Wang^2,3#^, Tao Luan^1,3^, Daxiang Chen^2,3^, Peiyu Huang^2,3^, Cheng Chen^1^, Xi Sun^2,3^, Qi Luo^2,3^, Jie Su^2,3^, Yunhui Zhang^1*^, Nanshan Zhong^1,2,3*^ and Zhongfang Wang^2,3*^

Correspondence to: wangzhongfang@gird.cn;

nanshan@vip.163.com;

yunhuizhang3188@126.com

**This PDF file includes:**

Figures. S1 to S5

Tables S1 to S5


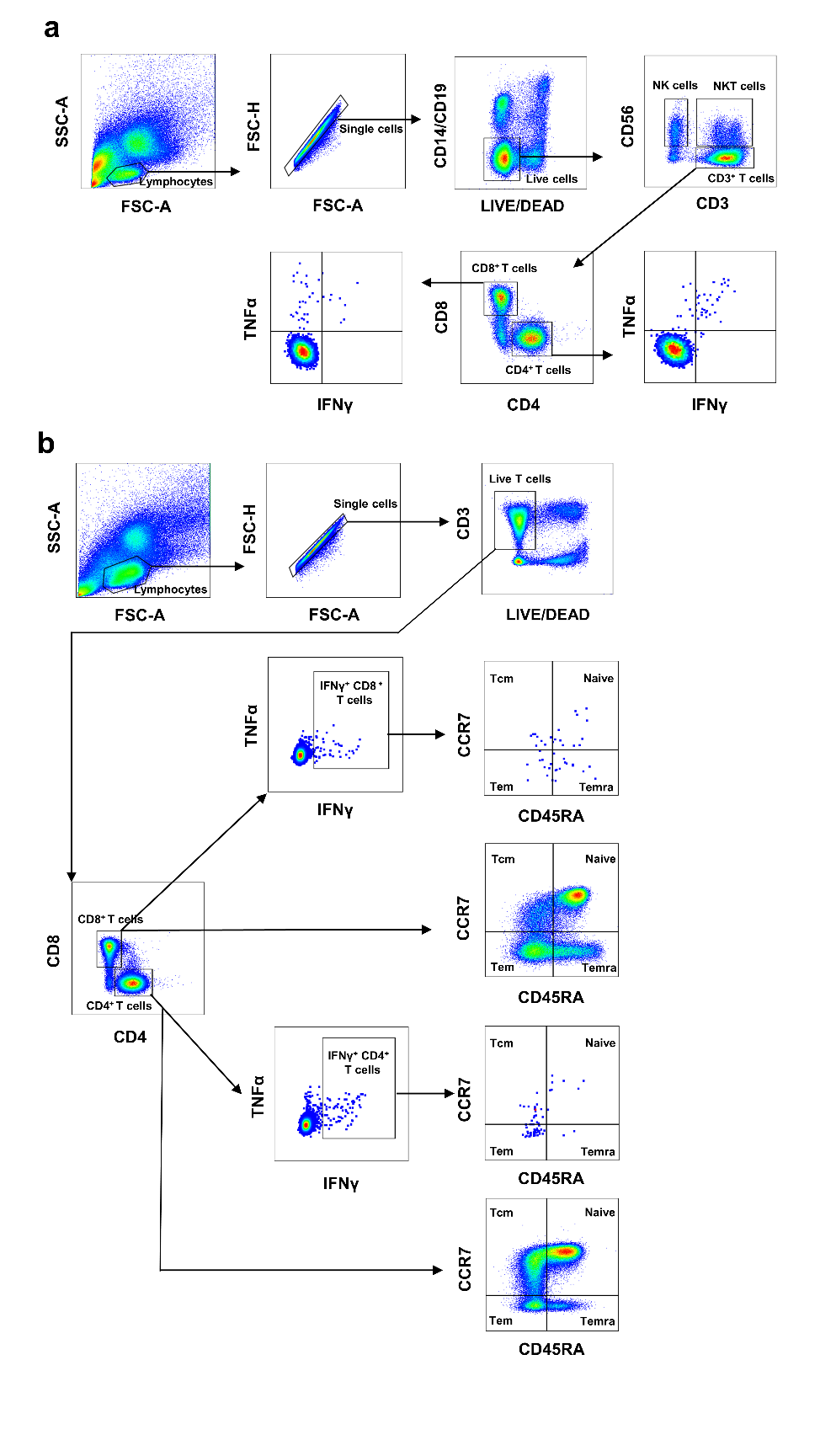


**Supplementary Figure 1. Gating strategy for flow cytometry experiments.** (**a** and **b**) Peripheral blood mononuclear cells (PBMCs) were stimulated with SARS-CoV-2 S-M-N-E peptides overnight and then immuno-stained for surface and intracellular. **a** Analysis of PBMCs in acute COVID-19. Live cells were gated from singlets derived from lymphocytes. Natural killer (NK), NKT and CD3^+^ T cells were further gated based on CD3 and CD56. CD4^+^ and CD8^+^ T cells from the CD3^+^ population were further gated for IFNγ and TNFα. **b** Analysis of paired PBMCs in the acute and convalescent phases. Live CD3^+^ T cells were gated from singlets derived from lymphocytes. Bulk and IFN^+^ CD4^+^ and CD8^+^ T cells were further divided into four memory phenotypes by surface markers CD45RA and CCR7.


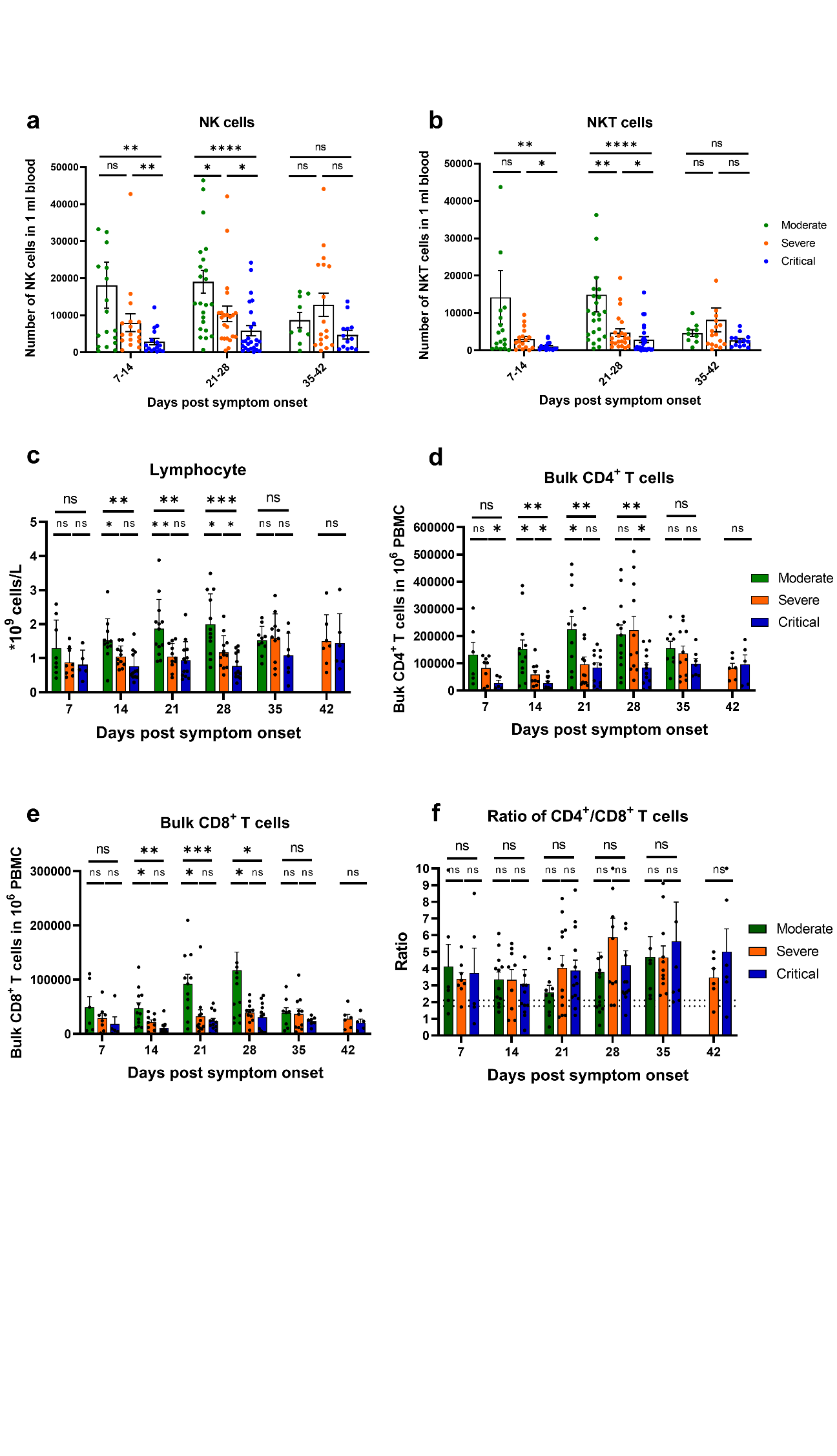


**Supplementary Figure 2. Analysis of lymphocytes, bulk T cells, NK and NKT cell populations during acute infection. a**–**b** Comparison of NK and NKT cell populations in moderate, severe, and critical groups during the early phase (7–14 dps), middle phase (21–28 dps), and late phase (35–42 dps) following acute SARS-CoV-2 infection (moderate, 7-14 dps n=19, 21-28 dps n=26, 35 dps n=10; severe, 7-14 dps n=19, 21-28 dps n=23, 35-42 dps n=18; critical, 7-14 dps n=18, 21-28 dps n=25, 35-42 dps n=13). **c** Comparison of absolute lymphocyte counts recorded in clinical data during hospitalization in the three groups during acute COVID-19. **d**–**f** Analysis of the number of bulk CD4^+^, CD8^+^ T cells, and the CD4/CD8 ratio in the three groups post acute infection. Comparisons were performed using Mann–Whitney tests. Each dot represents one donor. **P* < 0.05, ***P* < 0.01, ****P* < 0.001.


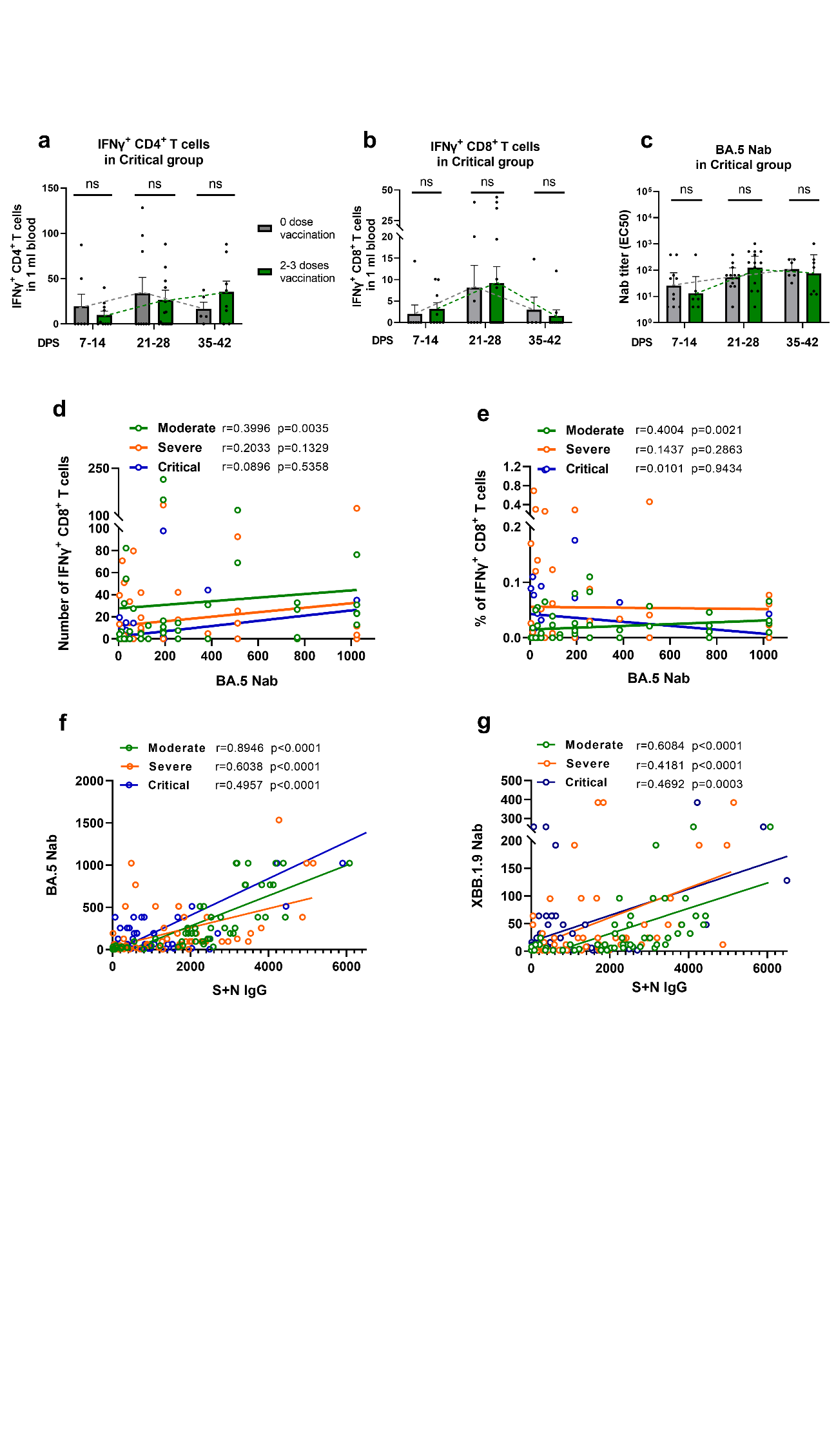


**Supplementary Figure 3. SARS-CoV-2-specific immune parameters comparison and correlation analysis. a-c** Comparison of the IFNγ^+^CD4^+^ and CD8^+^ T cell responses, as well as BA.5 Nab titers, during acute COVID-19 in 0 dose or 2-3 doses vaccinated critical patients. **d**–**e** The correlation between BA.5 Nab titers, and the number (**a**) and frequency (**b**) of IFNγ^+^CD8^+^ T cells in moderate (n=49), severe (n=56), and critical (n=50) groups. **f**–**g** The correlation of S+N IgG and BA.5 Nab (**c**) and XBB.1.9 (**d**) in moderate, severe, and critical groups. Spearman’s correlation coefficients and significance are shown accordingly. Each dot represents one donor. **P* < 0.05, ***P* < 0.01, ****P* < 0.001.

**
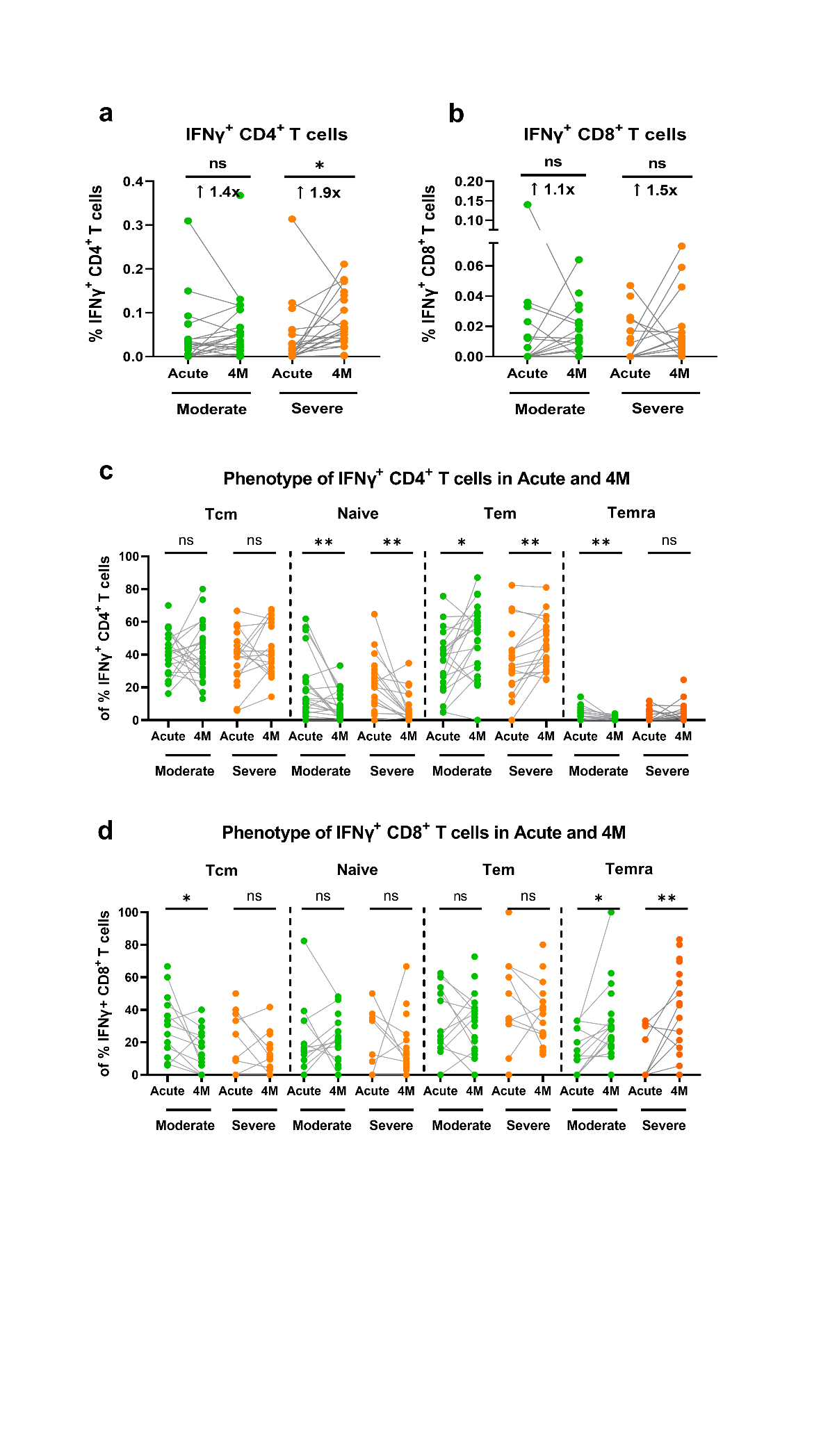
**

**Supplementary Figure 4. Phenotype transformation and frequency change of virus-specific T cells. a-b** Comparison of the frequency of virus-specific CD4^+^ and CD8^+^ T cells from the acute phase to 4M in the individuals recovered from moderate (n=25) and severe (n=24). **c**–**d** Analysis of the virus-specific CD4^+^ and CD8^+^ T cell phenotype transformation from acute infection to 4 months in the convalescence of moderate (n=25) and severe (n=24) COVID-19 patients, including central memory T cells (Tcm), effector memory T cells (Tem), terminally differentiated effector cells (Temra), and naïve T cells. Comparisons of paired samples were performed using the Wilcoxon matched-pairs signed rank test. Each dot represents one donor. **P* < 0.05, ***P* < 0.01, ****P* < 0.001.

**
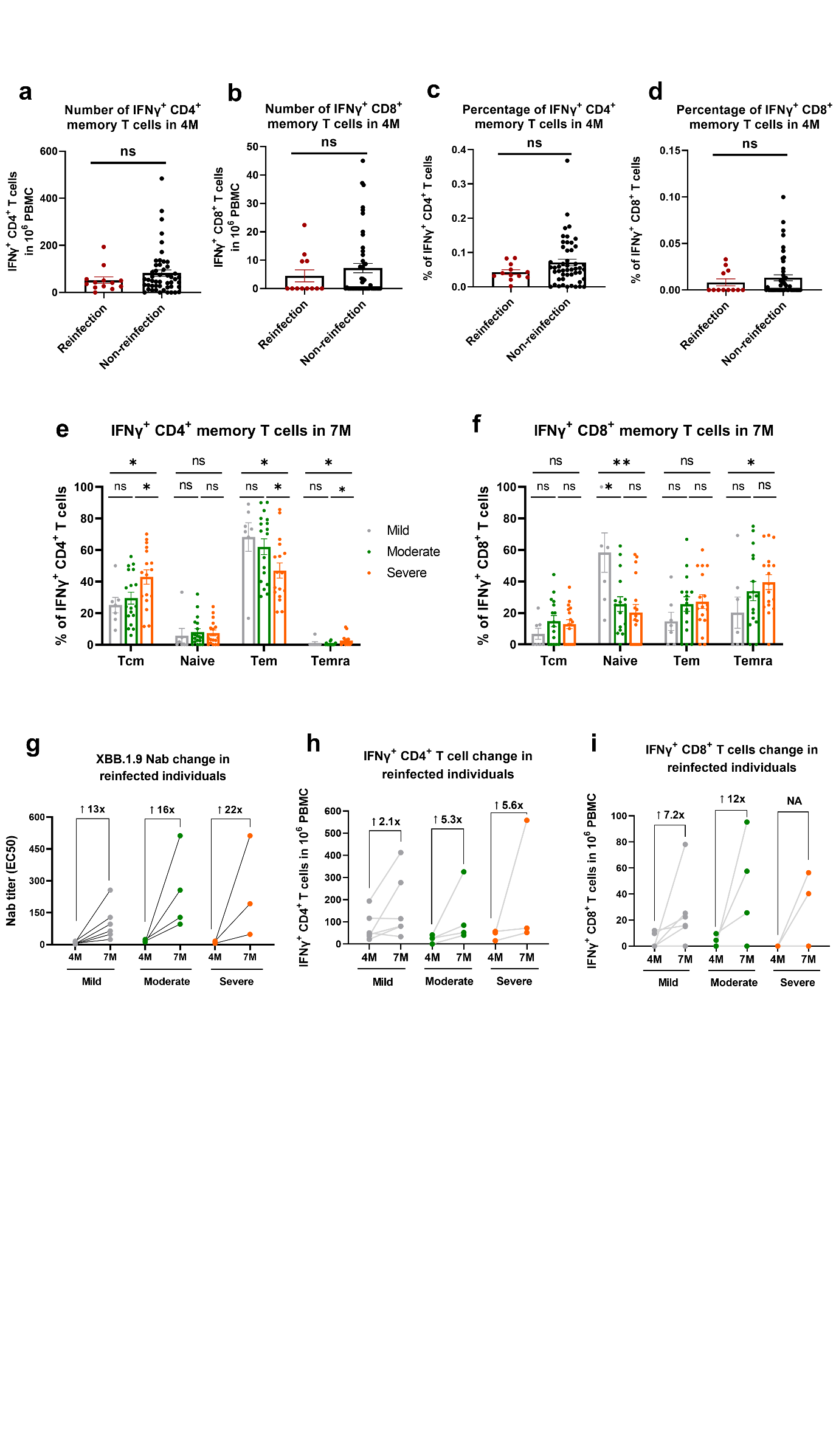
**

**Supplementary Figure 5. Long-term immune maintenance, and recall responses after reinfection. a-d** Comparison of the number (**a**,**b**) and percentage (**c**,**d**) of IFNγ^+^CD4^+^ and CD8^+^ T cell responses in 4M between individuals with (n=13) and without (n=49) subsequent reinfection. **e-f** Frequency of different virus-specific memory T cell phenotypes in the mild, moderate and severe groups in 7M. **g-i** Immune response recalls in individuals with serologically confirmed reinfection, including XBB.1.9 Nab (**g**), virus-specific CD4^+^ (**h**), or CD8^+^ T cells (**i**) in the mild (n=6), moderate (n=4), and severe (n=3) groups. Each dot represents one patient. Comparisons of paired samples were performed using the Wilcoxon matched-pairs signed rank test. Comparisons between groups were performed using Mann–Whitney tests. NA: not applicable. **P* < 0.05, ***P* < 0.01, ****P* < 0.001.

**Supplementary Table 1.** Clinical characteristics and demographic data of hospitalized COVID-19 patients.

|  | **Patient groups** | | | ***P* value** |
| --- | --- | --- | --- | --- |
|  | Moderate | Severe | Critical |  |
| Participants, no. | 47 | 45 | 30 | / |
| Basic characteristics |  | | | |
| Age (y) | 56.9 | 71.5 | 74.1 | <0.0001 |
| Male sex, no. (%) | 21 (44) | 37 (82) | 25 (83) | <0.0001 |
| BMI | 23.8 | 23.7 | 22.8 | 0.48 |
| Length of hospitalization | 8.4 | 12.3 | 22.1 | <0.0001 |
| Comorbidity, no. (%) |  | | | |
| COPD | 4 (8) | 9 (20) | 9 (30) | 0.052 |
| Bronchiectasis | 3 (6) | 1 (2) | 2 (6) | 0.57 |
| Diabetes | 6 (12) | 10 (22) | 9 (30) | 0.17 |
| Hypertension | 14 (29) | 18 (40) | 20 (66) | 0.006 |
| Hyperuricemia & Gout | 7 (14) | 6 (13) | 3 (10) | 0.82 |
| Cardiovascular disease | 10 (12) | 14 (31) | 16 (53) | 0.013 |
| Cancer | 1 (2) | 3 (6) | 3 (10) | 0.33 |
| Cerebral infarction | 1 (2) | 3 (6) | 10 (33) | <0.0001 |
| Chronic Renal disease | 2 (4) | 1 (2) | 3 (10) | 0.3 |
| Connective tissue disease | 3 (6) | 8 (17) | 8 (26) | 0.050 |
| Bacterial or fungal co-infection | 4 (8) | 12 (26) | 16 (53) | <0.0001 |
| Total number of comorbidities | 1.2 | 2.0 | 3.7 | <0.0001 |
| Clinical outcomes, no. (%) |  | | | |
| Thrombotic events | 1 (2) | 8 (17) | 14 (46) | <0.0001 |
| Death | 0 (0) | 0 (0) | 18 (60) | <0.0001 |

**Supplementary Table 2.** Detailed data for kinetic fitting of immune parameters.

| Figure No. | Group | Parameter | Peak time (dps) | Peak response | 95% Confidence Internal |
| --- | --- | --- | --- | --- | --- |
| Fig. 2c | Moderate | IFNγ^+^CD4^+^T cell | 24.1 | 145.21 | 84.45 to 205.97 |
|  | Severe |  | 23.3 | 150.13 | 84.04 to 230.23 |
|  | Critical |  | 28.0 | 46.50 | 23.77 to 69.22 |
| Fig. 2e | Moderate | IFNγ^+^CD8^+^T cell | 13.3 | 60.67 | 28.05 to 93.30 |
|  | Severe |  | 17.0 | 19.57 | 6.97 to 32.17 |
|  | Critical |  | 23.7 | 7.12 | 2.28 to 11.96 |
| Fig. 3a | Moderate | BA.5 Nab | 24.1 | 402.64 | 265.24 to 540.04 |
|  | Severe |  | 28.3 | 329.52 | 211.77 to 447.27 |
|  | Critical |  | 30.9 | 283.99 | 171.38 to 396.61 |
| Fig. 3d | Moderate | S+N IgG | 23.0 | 3.16 | 2.71 to 3.60 |
|  | Severe |  | 31.1 | 3.08 | 2.82 to 3.35 |
|  | Critical |  | 33.0 | 2.75 | 2.33 to 3.17 |
| Fig. 3e | Moderate | XBB.1.9 Nab | 22.8 | 50.88 | 25.77 to 76.00 |
|  | Severe |  | 26.2 | 67.41 | 31.57 to 103.24 |
|  | Critical |  | 34.8 | 91.59 | 49.00 to 134.19 |

**Supplementary Table 3.** COVID-19 Vaccination and pharmacological treatment of research cohorts.

|  | **Acute infection** | | | **Follow-up** | | |
| --- | --- | --- | --- | --- | --- | --- |
|  | Moderate | Severe | Critical | Mild | Moderate | Severe |
| Participants, no. | 47 | 45 | 30 | 16 | 26 | 26 |
| Vaccination information, no. (%) |  | | | | | |
| Vaccine brands | CoronaVac or BBIBP-CorV | | | CoronaVac or BBIBP-CorV | | |
| Average vaccine dose | 2.3 | 2.2 | 1.9 | 2.5 | 2.4 | 2.1 |
| Unvaccinated | 5 (11) | 6 (13) | 10 (33) | 1 (6) | 3 (12) | 4 (15) |
| 1 dose recipients | 0 (0) | 0 (0) | 1 (3) | 1 (6) | 2 (7) | 1 (4) |
| 2-3 doses recipients | 42 (89) | 39 (87) | 19 (63) | 14 (87) | 21 (81) | 21 (81) |
| Previous SARS-CoV-2 Infection  (pre-Omicron BA.5) | 0 | 0 | 0 | 0 | 0 | 0 |
| Steroids, no. (%) |  | | | | | |
| Prednisone | 2 (4) | 5 (11) | 7 (23) | 0 (0) | 0 (0) | 3 (12) |
| Dexamethasone | 3 (6) | 10 (22) | 2 (6) | 0 (0) | 0 (0) | 0 (0) |
| Methylprednisolone | 19 (40) | 22 (49) | 21 (70) | 0 (0) | 4 (15) | 8 (30) |
| Immunosuppressants,  no. (%) |  | | | | | |
| Hydroxychloroquine | 2 (4) | 0 (0) | 1 (3) | 0 (0) | 1 (4) | 0 (0) |
| Cyclosporine | 1 (2) | 0 (0) | 1 (3) | 0 (0) | 0 (0) | 0 (0) |
| Methotrexate | 0 (0) | 0 (0) | 0 (0) | 0 (0) | 1 (4) | 0 (0) |
| Tacrolimus | 0 (0) | 0 (0) | 1 (3) | 0 (0) | 0 (0) | 0 (0) |
| Mycophenolate mofetil | 0 (0) | 0 (0) | 1 (3) | 0 (0) | 0 (0) | 0 (0) |
| Antiviral drugs, no. (%) |  |  |  |  |  |  |
| Tocilizumab | 1 (2) | 1 (2) | 0 (0) | 0 (0) | 0 (0) | 0 (0) |
| Nirmatrelvir/Ritonavir | 2 (4) | 8 (18) | 8 (26) | 0 (0) | 0 (0) | 0 (0) |
| Molnupiravir | 0 (0) | 0 (0) | 3 (10) | 0 (0) | 0 (0) | 0 (0) |
| Azvudine | 2 (4) | 5 (11) | 4 (13) | 0 (0) | 0 (0) | 2 (8) |
| Ganciclovir | 0 (0) | 0 (0) | 1 (3) | 0 (0) | 0 (0) | 0 (0) |
| Oseltamivir | 2 (4) | 0 (0) | 0 (0) | 0 (0) | 0 (0) | 0 (0) |

**Supplementary Table 4.** Follow-up information.

|  | **Patient groups** | | | | | |
| --- | --- | --- | --- | --- | --- | --- |
|  | Mild | | Moderate | | Severe | |
| Participants, no. | 16 | | 26 | | 26 | |
| Overlapping with acute infected cohort | 0 (0) | | 12 (46) | | 13 (50) | |
| Follow-up time from symptom onset |  | | | | | |
| First visit | 117 (80-131) | | 116 (89-128) | | 115 (86-128) | |
| Second visit | 220 (182-234) | | 219 (192-231) | | 218 (189-231) | |
| Re-infection with symptomatic SARS-COV-2, no. (%) |  | | | | | |
| Up to the first visit | 0 (0) | | 0 (0) | | 0 (0) | |
| Between first and second follow-up | 4 (25) | | 3 (11) | | 2 (7) | |
| Acute phase paired samples from symptom onset (range) | / | | 20.8 (9-34) | | 24.3 (8-35) | |
| Long COVID-19 symptoms, no. (%) | mean days | >90d | mean days | >90d | mean days | >90d |
| Cough & Expectoration | 29 | 1 (6) | 44 | 8 (30) | 59 | 7 (28) |
| Dyspnea | 3 | 0 (0) | 62 | 9 (34) | 113 | 15 (60) |
| Palpitation | 2 | 0 (0) | 42 | 6 (23) | 41 | 5 (20) |
| Fatigue | 9 | 0 (0) | 114 | 16 (61) | 110 | 15 (60) |
| Nausea & Anorexia | 14 | 1 (6) | 38 | 5 (19) | 37 | 6 (24) |
| muscle pain and weakness | 2 | 0 (0) | 66 | 9 (34) | 55 | 7 (28) |
| Headache | 2 | 0 (0) | 17 | 4 (15) | 24 | 3 (12) |
| Loss of smell and taste | 3 | 0 (0) | 43 | 6 (23) | 52 | 7 (28) |
| Impaired cognitive function | 4 | 0 (0) | 102 | 13 (50) | 82 | 10 (40) |
| Insomnia and early awakening | 3 | 0 (0) | 46 | 7 (26) | 61 | 8 (32) |
| Anxiety and emotional instability | 2 | 0 (0) | 22 | 3 (11) | 25 | 3 (12) |
| Arthralgia | 0 | 0 (0) | 21 | 3 (11) | 41 | 6 (24) |

**Supplementary Table 5.** Detailed statistical information for non-parametric data.

| **Figure No.** | **Parameter** | **Group** | **dps** | **25% Percentile** | **Median** | **75% Percentile** | **IQR** |
| --- | --- | --- | --- | --- | --- | --- | --- |
| Fig. 2b | IFNγ^+^ CD4^+^  T cells (number in 1 ml blood) | Moderate | 7 | 4.375 | 51.85 | 182.6 | 178.225 |
|  |  |  | 14 | 2.15 | 15.2 | 84 | 81.85 |
|  |  |  | 21 | 19.5 | 138.1 | 248.4 | 228.9 |
|  |  |  | 28 | 0.25 | 36.9 | 144.2 | 143.95 |
|  |  |  | 35 | 0.7 | 33 | 73.4 | 72.7 |
|  |  | Severe | 7 | 1.05 | 19.85 | 37.43 | 36.38 |
|  |  |  | 14 | 0 | 13.9 | 49.9 | 49.9 |
|  |  |  | 21 | 19.3 | 34.8 | 157.1 | 137.8 |
|  |  |  | 28 | 11.03 | 57.85 | 224 | 212.97 |
|  |  |  | 35 | 3.2 | 14 | 169.5 | 166.3 |
|  |  |  | 42 | 4.425 | 37.1 | 210.3 | 205.875 |
|  |  | Critical | 7 | 0 | 0 | 8.45 | 8.45 |
|  |  |  | 14 | 0 | 0 | 9.25 | 9.25 |
|  |  |  | 21 | 0 | 0 | 48.6 | 48.6 |
|  |  |  | 28 | 0 | 16 | 81.83 | 81.83 |
|  |  |  | 35 | 12.6 | 14.8 | 44.2 | 31.6 |
|  |  |  | 42 | 0 | 19 | 47.98 | 47.98 |
| Fig. 2d | IFNγ^+^ CD8^+^  T cells (number in 1 ml blood) | Moderate | 7 | 0 | 0 | 62.65 | 62.65 |
|  |  |  | 14 | 6.75 | 18.9 | 69.35 | 62.6 |
|  |  |  | 21 | 0 | 4.9 | 68.9 | 68.9 |
|  |  |  | 28 | 0 | 8.8 | 27.45 | 27.45 |
|  |  |  | 35 | 0 | 1.4 | 11.55 | 11.55 |
|  |  | Severe | 7 | 0 | 0 | 32.88 | 32.88 |
|  |  |  | 14 | 0 | 0 | 42.2 | 42.2 |
|  |  |  | 21 | 0 | 0 | 41.05 | 41.05 |
|  |  |  | 28 | 0 | 0 | 25.3 | 25.3 |
|  |  |  | 35 | 0 | 0 | 17.3 | 17.3 |
|  |  |  | 42 | 0 | 0 | 8.125 | 8.125 |
|  |  | Critical | 7 | 0 | 0 | 6 | 6 |
|  |  |  | 14 | 0 | 0 | 2.6 | 2.6 |
|  |  |  | 21 | 0 | 0 | 1.15 | 1.15 |
|  |  |  | 28 | 0 | 0 | 9.6 | 9.6 |
|  |  |  | 35 | 0 | 0 | 3.7 | 3.7 |
|  |  |  | 42 | 0 | 0 | 0 | 0 |
| Fig. 2f | IFNγ^+^ CD4^+^  T cells (number in 1 ml blood) | Short-term hospitalization | 7-14 | 4.3 | 20.8 | 83 | 78.7 |
|  |  |  | 21-28 | 0.25 | 56.3 | 175.9 | 175.65 |
|  |  |  | 35-42 | 2.75 | 24.25 | 76.38 | 73.63 |
|  |  | Extended hospitalization | 7-14 | 0 | 0 | 6.875 | 6.875 |
|  |  |  | 21-28 | 0 | 21.3 | 75.9 | 75.9 |
|  |  |  | 35-42 | 8.35 | 14.8 | 61.95 | 53.6 |
| Fig. 2g | IFNγ^+^ CD8^+^  T cells (number in 1 ml blood) | Short-term hospitalization | 7-14 | 0 | 4.8 | 27.5 | 27.5 |
|  |  |  | 21-28 | 0 | 4.45 | 29.43 | 29.43 |
|  |  |  | 35-42 | 0 | 0.7 | 11.6 | 11.6 |
|  |  | Extended hospitalization | 7-14 | 0 | 0 | 4.1 | 4.1 |
|  |  |  | 21-28 | 0 | 0 | 7.3 | 7.3 |
|  |  |  | 35-42 | 0 | 0 | 0 | 0 |
| Fig. 3b | BA.5 Nab titer | Moderate | 7 | 4 | 128 | 384 | 380 |
|  |  |  | 14 | 17 | 128 | 352 | 335 |
|  |  |  | 21 | 36 | 192 | 768 | 732 |
|  |  |  | 28 | 64 | 256 | 768 | 704 |
|  |  |  | 35 | 44 | 112 | 336 | 292 |
|  |  | Severe | 7 | 4 | 8 | 120 | 116 |
|  |  |  | 14 | 4 | 32 | 96 | 92 |
|  |  |  | 21 | 24 | 72 | 416 | 392 |
|  |  |  | 28 | 60 | 160 | 256 | 196 |
|  |  |  | 35 | 48 | 96 | 512 | 464 |
|  |  |  | 42 | 16 | 96 | 384 | 368 |
|  |  | Critical | 7 | 4 | 12 | 32 | 28 |
|  |  |  | 14 | 6 | 12 | 224 | 218 |
|  |  |  | 21 | 10 | 128 | 354 | 344 |
|  |  |  | 28 | 64 | 96 | 384 | 320 |
|  |  |  | 35 | 16 | 128 | 256 | 240 |
|  |  |  | 42 | 27 | 128 | 208 | 181 |
| Fig. 3d | S+N IgG titer | Moderate | 7 | 54.15 | 2439 | 3726 | 3671.85 |
|  |  |  | 14 | 358.3 | 2181 | 2565 | 2206.7 |
|  |  |  | 21 | 797.6 | 2338 | 3496 | 2698.4 |
|  |  |  | 28 | 1006 | 2128 | 3166 | 2160 |
|  |  |  | 35 | 437.3 | 1392 | 3175 | 2737.7 |
|  |  | Severe | 7 | 57.08 | 431.4 | 1207 | 1149.92 |
|  |  |  | 14 | 23.22 | 306 | 1980 | 1956.78 |
|  |  |  | 21 | 249.6 | 1231 | 2832 | 2582.4 |
|  |  |  | 28 | 262.6 | 1904 | 3178 | 2915.4 |
|  |  |  | 35 | 479 | 1697 | 2872 | 2393 |
|  |  |  | 42 | 586.8 | 1284 | 2033 | 1446.2 |
|  |  | Critical | 7 | 5.555 | 481.4 | 1039 | 1033.445 |
|  |  |  | 14 | 101 | 173.7 | 1062 | 961 |
|  |  |  | 21 | 76.57 | 1049 | 2251 | 2174.43 |
|  |  |  | 28 | 104.2 | 775.1 | 2289 | 2184.8 |
|  |  |  | 35 | 193.2 | 426.8 | 1534 | 1340.8 |
|  |  |  | 42 | 124.8 | 549.9 | 1173 | 1048.2 |
| Fig. 3f | XBB Nab titer | Moderate | 7 | 2 | 22 | 60 | 58 |
|  |  |  | 14 | 3 | 10 | 51 | 48 |
|  |  |  | 21 | 6 | 8 | 48 | 42 |
|  |  |  | 28 | 6 | 12 | 28 | 22 |
|  |  |  | 35 | 2 | 12 | 36 | 34 |
|  |  | Severe | 7 | 2 | 2 | 12 | 10 |
|  |  |  | 14 | 2 | 4 | 24 | 22 |
|  |  |  | 21 | 6.5 | 20 | 88 | 81.5 |
|  |  |  | 28 | 12 | 24 | 84 | 72 |
|  |  |  | 35 | 6 | 12 | 48 | 42 |
|  |  |  | 42 | 2 | 12 | 24 | 22 |
|  |  | Critical | 7 | 2 | 2 | 11 | 9 |
|  |  |  | 14 | 2 | 4 | 56 | 54 |
|  |  |  | 21 | 5 | 12 | 80 | 75 |
|  |  |  | 28 | 3 | 18 | 60 | 57 |
|  |  |  | 35 | 6 | 24 | 256 | 250 |
|  |  |  | 42 | 2 | 8 | 25 | 23 |
| Fig. 3g | BA.5 Nab titer | Short-term hospitalization | 7-14 | 7 | 56 | 208 | 201 |
|  |  |  | 21-28 | 96 | 192 | 576 | 480 |
|  |  |  | 35-42 | 48 | 96 | 352 | 304 |
|  |  | Extended hospitalization | 7-14 | 4 | 12 | 64 | 60 |
|  |  |  | 21-28 | 24 | 64 | 352 | 328 |
|  |  |  | 35-42 | 24 | 128 | 256 | 232 |
| Fig. 4b | BA.5 Nab titer | Mild | 4M | 32 | 48 | 96 | 64 |
|  |  | Moderate | Acute | 36 | 192 | 352 | 316 |
|  |  |  | 4M | 48 | 96 | 192 | 144 |
|  |  | Severe | Acute | 26 | 96 | 256 | 230 |
|  |  |  | 4M | 40 | 112 | 352 | 312 |
| Fig. 4c | WT Nab titer | Mild | 4M | 96 | 128 | 384 | 288 |
|  |  | Moderate | Acute | 96 | 512 | 1024 | 928 |
|  |  |  | 4M | 104 | 256 | 512 | 408 |
|  |  | Severe | Acute | 13 | 256 | 768 | 755 |
|  |  |  | 4M | 12 | 128 | 480 | 468 |
| Fig. 4d | XBB Nab titer | Mild | 4M | 3 | 6 | 12 | 9 |
|  |  | Moderate | Acute | 6.5 | 18 | 48 | 41.5 |
|  |  |  | 4M | 12 | 16 | 48 | 36 |
|  |  | Severe | Acute | 9 | 16 | 48 | 39 |
|  |  |  | 4M | 12 | 16 | 48 | 36 |
| Fig. 4e | S+N IgG titer | Mild | 4M | 398.5 | 561.5 | 839.6 | 441.1 |
|  |  | Moderate | Acute | 542.8 | 2078 | 3346 | 2803.2 |
|  |  |  | 4M | 432.5 | 906.8 | 1797 | 1364.5 |
|  |  | Severe | Acute | 250 | 1093 | 2248 | 1998 |
|  |  |  | 4M | 47.39 | 346.7 | 1213 | 1165.61 |
| Fig. 5b | IFNγ^+^ CD4^+^  T cells in 10^6^ PBMCs | Mild | 4M | 21.13 | 37.85 | 52.8 | 31.67 |
|  |  | Moderate | 4M | 24.7 | 42.7 | 103.2 | 78.5 |
|  |  | Severe | 4M | 32.4 | 64.3 | 117.5 | 85.1 |
|  | IFNγ^+^ CD8^+^  T cells in 10^6^ PBMCs | Mild | 4M | 0 | 0 | 2.4 | 2.4 |
|  |  | Moderate | 4M | 0 | 3 | 9.65 | 9.65 |
|  |  | Severe | 4M | 0 | 0.8 | 16.18 | 16.18 |
| Fig. 5c | IFNγ^+^ CD4^+^  T cells (Frequency) | Moderate | Acute | 0 | 0.018 | 0.0395 | 0.0395 |
|  |  |  | 4M | 0.02125 | 0.042 | 0.06425 | 0.043 |
|  |  | Severe | Acute | 0 | 0.0165 | 0.045 | 0.045 |
|  |  |  | 4M | 0.02575 | 0.0595 | 0.1233 | 0.09755 |
|  | IFNγ^+^ CD8^+^  T cells (Frequency) | Moderate | Acute | 0 | 0 | 0.0105 | 0.0105 |
|  |  |  | 4M | 0 | 0.0045 | 0.02025 | 0.02025 |
|  |  | Severe | Acute | 0 | 0 | 0.012 | 0.012 |
|  |  |  | 4M | 0 | 0.002 | 0.01275 | 0.01275 |
| Fig. 5d | Bulk CD4^+^ T cells in 10^6^ PBMCs | Mild | 4M | 44428 | 83637 | 145385 | 100957 |
|  |  | Moderate | Acute | 17767 | 45078 | 108147 | 90380 |
|  |  |  | 4M | 82191 | 105214 | 143147 | 60956 |
|  |  | Severe | Acute | 9723 | 20853 | 40834 | 31111 |
|  |  |  | 4M | 62215 | 109212 | 164407 | 102192 |
|  | Bulk CD8^+^ T cells in 10^6^ PBMCs | Mild | 4M | 41567 | 59527 | 69270 | 27703 |
|  |  | Moderate | Acute | 5660 | 19711 | 31687 | 26027 |
|  |  |  | 4M | 28147 | 45242 | 72595 | 44448 |
|  |  | Severe | Acute | 2206 | 6526 | 11673 | 9467 |
|  |  |  | 4M | 35568 | 58235 | 99998 | 64430 |
| Fig. 6c | IFNγ^+^ CD4^+^  T cells in 10^6^ PBMCs | Mild | 7M | 5.775 | 17.75 | 42.6 | 36.825 |
|  |  | Moderate | 7M | 18.7 | 56.25 | 87.05 | 68.35 |
|  |  | Severe | 7M | 27.45 | 70.6 | 111 | 83.55 |
| Fig. 6d | IFNγ^+^ CD8^+^  T cells in 10^6^ PBMCs | Mild | 7M | 0 | 0 | 5.4 | 5.4 |
|  |  | Moderate | 7M | 0 | 0 | 4.05 | 4.05 |
|  |  | Severe | 7M | 0 | 3.5 | 12.8 | 12.8 |
| Fig. 6e | XBB Nab titer | Mild | 7M | 2 | 10 | 33 | 31 |
|  |  | Moderate | 7M | 3.5 | 6 | 12 | 8.5 |
|  |  | Severe | 7M | 4 | 6 | 14 | 10 |
